# Using toponomics to characterize phenotypic diversity in alveolar macrophages from male mice treated with exogenous SP-A1

**DOI:** 10.1186/s40364-019-0181-z

**Published:** 2020-02-13

**Authors:** David S. Phelps, Vernon M. Chinchilli, Judith Weisz, Debra Shearer, Xuesheng Zhang, Joanna Floros

**Affiliations:** 1 Penn State Center for Host defense, Inflammation, and Lung Disease (CHILD) Research and Departments of Pediatrics, The Pennsylvania State University College of Medicine, Hershey, PA 17033 USA; 2 Public Health Sciences; and Obstetrics and Gynecology, The Pennsylvania State University College of Medicine, Hershey, PA 17033 USA; 3 Obstetrics and Gynecology, The Pennsylvania State University College of Medicine, Hershey, PA 17033 USA

**Keywords:** Alveolar macrophage, Activation, Immunophenotype, Surfactant, Phenotype, Interactome, Heterogeneity, Diversity, Single cell analysis, Biomarker

## Abstract

**Background:**

We used the Toponome Imaging System (TIS) to identify “patterns of marker expression”, referred to here as combinatorial molecular phenotypes (CMPs) in alveolar macrophages (AM) in response to the innate immune molecule, SP-A1.

**Methods:**

We compared 114 AM from male SP-A deficient mice. One group (*n* = 3) was treated with exogenous human surfactant protein A1 (hSP-A1) and the other with vehicle (*n* = 3). AM obtained by bronchoalveolar lavage were plated onto slides and analyzed using TIS to study the AM toponome, the spatial network of proteins within intact cells. With TIS, each slide is sequentially immunostained with multiple FITC-conjugated antibodies. Images are analyzed pixel-by-pixel identifying all of the proteins within each pixel, which are then designated as CMPs. CMPs represent organized protein clusters postulated to contribute to specific functions.

**Results:**

1) We compared identical CMPs in KO and SP-A1 cells and found them to differ significantly (*p* = 0.0007). Similarities between pairs of markers in the two populations also differed significantly (*p* < 0.0001). 2) Focusing on the 20 most abundant CMPs for each cell, we developed a method to generate CMP “signatures” that characterized various groups of cells. Phenotypes were defined as cells exhibiting similar signatures of CMPs. i) AM were extremely diverse and each group contained cells with multiple phenotypes. ii) Among the 114 AM analyzed, no two cells were identical. iii) However, CMP signatures could distinguish among cell subpopulations within and between groups. iv) Some cell populations were enriched with SP-A1 treatment, some were more common without SP-A1, and some seemed not to be influenced by the presence of SP-A1. v) We also found that AM were more diverse in mice treated with SP-A1 compared to those treated with vehicle.

**Conclusions:**

AM diversity is far more extensive than originally thought. The increased diversity of SP-A1-treated mice points to the possibility that SP-A1 enhances or activates several pathways in the AM to better prepare it for its innate immune functions and other functions shown previously to be affected by SP-A treatment. Future studies may identify key protein(s) responsible for CMP integrity and consequently for a given function, and target it for therapeutic purposes.

## Background

The AM is the principal effector cell of innate immunity (the first line of host defense) in the lung. Its many functions are subject to complex regulation through autocrine [1] and paracrine mechanisms, as well as environmental factors [2], and other cell types, such as type II alveolar epithelial cells [3], which are not only responsible for the production of surfactant, a complex of proteins and lipids which is essential for lung function, but also produce a number of immunoregulatory molecules [4]. There is a large body of evidence demonstrating that the surfactant constituent, surfactant protein A (SP-A) has a profound regulatory effect on the AM, including regulating proinflammatory cytokine production, enhancing phagocytosis, and influencing actin metabolism [5–7]. This multi-faceted regulatory pattern may be one of the reasons for the well-documented heterogeneity of AM in the lung [7–10]. A model formulated some years ago described macrophages as being either classically (M1) or alternatively (M2) activated [11]. In recent years additional phenotypes have been added [12] and evidence suggests that phenotypes encompass a whole spectrum between the M1 and M2 extremes [13, 14]. Furthermore, macrophages from normal individuals may not have either M1 or M2 characteristics, and there are cases where a macrophage under some conditions may express M1 and M2 traits simultaneously [15]. The full functional significance of these phenotypic differences in AM remains to be determined.

SP-A, either alone (i.e. as an opsonin) or via its interaction with the AM, plays an important role in innate immunity and host defense. The importance of these actions of SP-A is clearly seen in the increased susceptibility to infection and decreased survival in mice lacking SP-A (SP-A knockout, KO) [16–18], which appear to have more vigorous, but poorly controlled reactions to a variety of potentially damaging stimuli, suggesting that normal AM regulation and function is disrupted when SP-A is absent [19, 20]. Both survival and SP-A rescue of the AM proteome exhibited sex-specific differences [18, 21]. Moreover, a single dose of SP-A in KO mice resulted in an AM proteome similar to that of the wild type (WT) mouse [5, 22] and phagocytosis and clearance of group B streptococcus in KO mice was increased when exogenous SP-A was co-administered with the bacteria [23]. In humans, however, unlike in rodents, there are two genes, *Sftpa1* and *Sftpa2*, encoding SP-A1 and SP-A2, respectively, and these two gene products have been shown to have a differential impact on several AM functions. These functions include bacterial phagocytosis and cytokine production by AM [18, 24, 25], actin polymerization in the AM [7], and effects on the AM proteome and miRNome [26–28]. Moreover, the effects of SP-A variants on the regulation of the AM proteome and miRNome, survival, and pulmonary mechanics after infection, vary with sex [26–30].

Although various approaches (proteomics, miRNAs, etc) have been used to attempt to characterize macrophage phenotype, the data derived from these approaches are averages of potentially complex cell populations and cannot characterize subpopulations unless combined with some type of cell fractionation prior to analysis. Flow cytometry [31], and more recently cytometry TOF (time-of-flight) [32], have enabled single cell immunophenotyping of lung macrophages. Both of these methods using intact cells have advanced this field and documented macrophage heterogeneity but provide no data about localization of markers in the cell. However, flow cytometry, despite being able to detect as many as 20 different colors [33, 34], is based on whole cell data and does not provide any information with regards to co-localization within the cell and/or subcellular compartments.

**Fig. 1:**
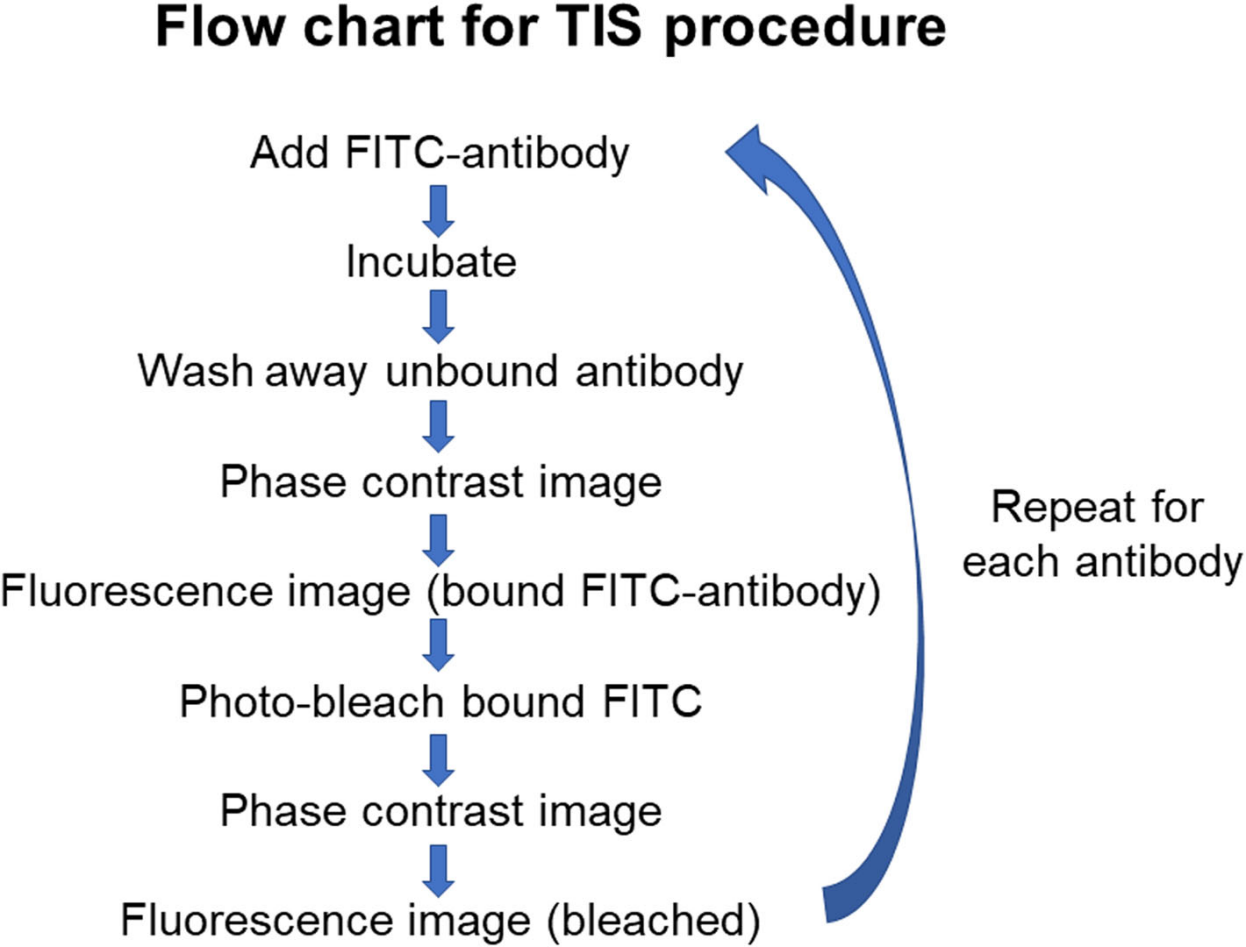
Flow Chart of TIS procedure. The basic steps of the TIS procedure are outlined

We have employed a powerful technology, the Toponome Imaging System (TIS™), also known as Imaging Cycler Microscopy, or Multi-epitope ligand cartography (MELC) to study the expression of multiple markers in intact, individual cells (as opposed to other technologies, such as proteomics, where cells are disrupted) and explore the SP-A:AM relationship. Several relevant TIS-related terms are defined in Table 1 and a flow chart of the procedure is shown in Fig. 1. Although prior studies with TIS, a serial immunostainer, have been largely descriptive and involved detailed analysis of one or two samples, some have done some limited comparisons of whole images of tissue sections [35–40]. In this study we investigated the effect of SP-A on AM phenotype using TIS. Towards this, we developed methods that allowed us to compare, for the first time, the expression of 13 markers in individual cells (114 AM) obtained from 6 different subjects, to study the effect of SP-A on AM phenotype.

**Table 1:** Glossary of TIS terminology

	Glossary of TIS terms
toponome	the spatial network of proteins within intact cells
TIS	toponome imaging system – a robotically-controlled serial immunostainer
CMP	combinatorial molecular phenotype- a designation that summarizes the presence [1] or absence (0) of all markers in a given pixel (i.e. 1000110100011)
binarization	selecting of a threshold in aligned, background-subtracted images of immuno-fluorescence above which a marker is considered present [1] and below which it is absent (0)
.xml file	a data file merging all of the binarization data from each marker into a single file and showing the CMP present in every pixel of an image
triplets	CMPs that are present in all three subjects in each experimental group in the 50 most frequent/abundant CMPs in the xml files.
2-of-3	CMPs that are present in 2 out of the 3 subjects in each group in the 50 most frequent/abundant CMPs in the xml files

Here we applied TIS (or MELC) [36, 41–44] to use Combinatorial Molecular Phenotypes or CMPs to characterize the heterogeneity of AM. A CMP is a designation indicating the presence or absence of all markers in a given pixel. In all images there are 2^n^ possible CMPs where *n* = the number of markers used. TIS is a high throughput robotically-controlled microscopic system developed by Schubert [36, 41–43]. It enables immunophenotyping cells in their native environment by using robotically controlled reiterative cycles of immunostaining (tagging), imaging, and photobleaching of FITC-conjugated antibodies. By applying dedicated software to the computerized images, TIS enables visualizing at the cellular and subcellular levels, not only the co-localization of proteins, but also their assembly to form protein clusters or supramolecular structures, and to enumerate them based on their CMPs. The significance of this approach is that proteins rarely function in isolation and their function often depends on the other proteins in their immediate proximity as members of a multiprotein complex. TIS provides this type of information by showing the pixel-by-pixel localization of multiple markers. Thus TIS, does not simply co-localize proteins, but via CMPs, enables identifying and enumerating supramolecular structures formed by protein clusters and represented by CMPs. Importantly, with TIS it is possible to identify even small subpopulations of cells in their native microenvironment. There is already evidence that physiological and pathological conditions result in differences in both the number and composition of CMPs and these conditions may be identified by CMPs with a unique composition [35, 36, 45].

Toponomics, like other “omics” technology can discover major gaps in our knowledge and provides the foundation for testable hypotheses and identification of biomarkers. Moreover, with TIS one can build on proteomics data to learn how molecules are organized within the crowded molecular space of cells and how these different molecules cooperate in time and space to bring about a specific cellular function [46]. Thus, TIS may provide the first steps toward translational research and/or therapeutic interventions [43]. Here, using TIS we investigated the differences between groups by comparing the presence of identical CMPs in these groups. We also compared these groups by assessing the similarities in the expression of markers making up the CMPs. Furthermore, we used CMP signatures that summarized the CMP content of individual cells to distinguish cell populations both within a given group and between two different groups.

## Methods

### Animals

Male SP-A KO mice on the C57BL6/J genetic background were used at 8–12 weeks of age. The mice were propagated and raised in our breeding colony at the Penn State College of Medicine. All mice were maintained under pathogen-free conditions or in barrier facilities with free access to food and water. Sentinel animals housed in the same animal rooms had no evidence of respiratory pathogens. This study was approved by the Institutional Animal Care and Use Committee of the Penn State College of Medicine.

### Treatment of mice with exogenous SP-A1

For these experiments mice were anesthetized by injection with Ketamine (Ketaject, Phoenix Pharmaceuticals Inc., St. Joseph, MO) and Xylazine (XYLA-JECT, Phoenix Pharmaceuticals Inc., St. Joseph, MO). SP-A1 was purified from stably transfected CHO cells and isolated by mannose affinity chromatography as described previously [25]. SP-A1 preparations were made with the SP-A1 6A^2^ variant. This is an SP-A1 variant that occurs in the general population with the greatest frequency [47, 48]. The exogenous SP-A1 preparation contained SP-A1 (10 μg) in 50 μl of sterile saline with 1 mM CaCl_2_. We have used this dose of exogenous SP-A in previous rescue studies [26]. Control animals received 50 μl of vehicle (saline and 1 mM CaCl_2_) alone. Anesthetized mice were suspended by their maxillary incisors, the bolus containing SP-A1 or vehicle placed in the pharynx, and the nostrils briefly blocked, resulting in aspiration of the bolus. The mice were returned to their cages after recovery from anesthesia. In previous studies [21, 22, 26] we have found this method to be very consistent and reproducible for introducing SP-A (and other fluids) to the lungs.

### Sample preparation

Eighteen hours after SP-A1 treatment the mice were euthanized and subjected to bronchoalveolar lavage (BAL) with phosphate-buffered saline (PBS), 1 mM EDTA to obtain AM which were washed and counted. Samples were prepared by placing a 0.5 mm thick plastic sheet in which a circular opening with a diameter of 8 mm was cut onto a microscope slide. An aliquot containing 100,000 cells was placed in the resulting well in a volume of 100 μl of serum-free RPMI medium. The cell compartment was covered with a plastic cap to limit evaporation and the slide was placed in the incubator for 45–60 min to allow the cells to adhere. At the conclusion of the attachment period the slides were gently washed by dipping them in PBS. The slides were then air dried (15 min), immersed in acetone at room temperature (10 s), then in hexanes that had been chilled to − 70 °C in a methanol/dry ice slush (90 s). The slides were stored at − 80 °C until used for TIS.

On the day TIS was performed, each slide was warmed to room temperature. A 1.0 mm thick rubber ring with a diameter of 10 mm was placed over the cells. The cells were rehydrated, treated with normal goat serum diluted 1:50 with PBS for 1 h, and washed repeatedly with PBS. The slide was then placed on the microscope in the TIS chamber and a view field selected.

### Toponome imaging system (TIS)

**Fig. 2:**
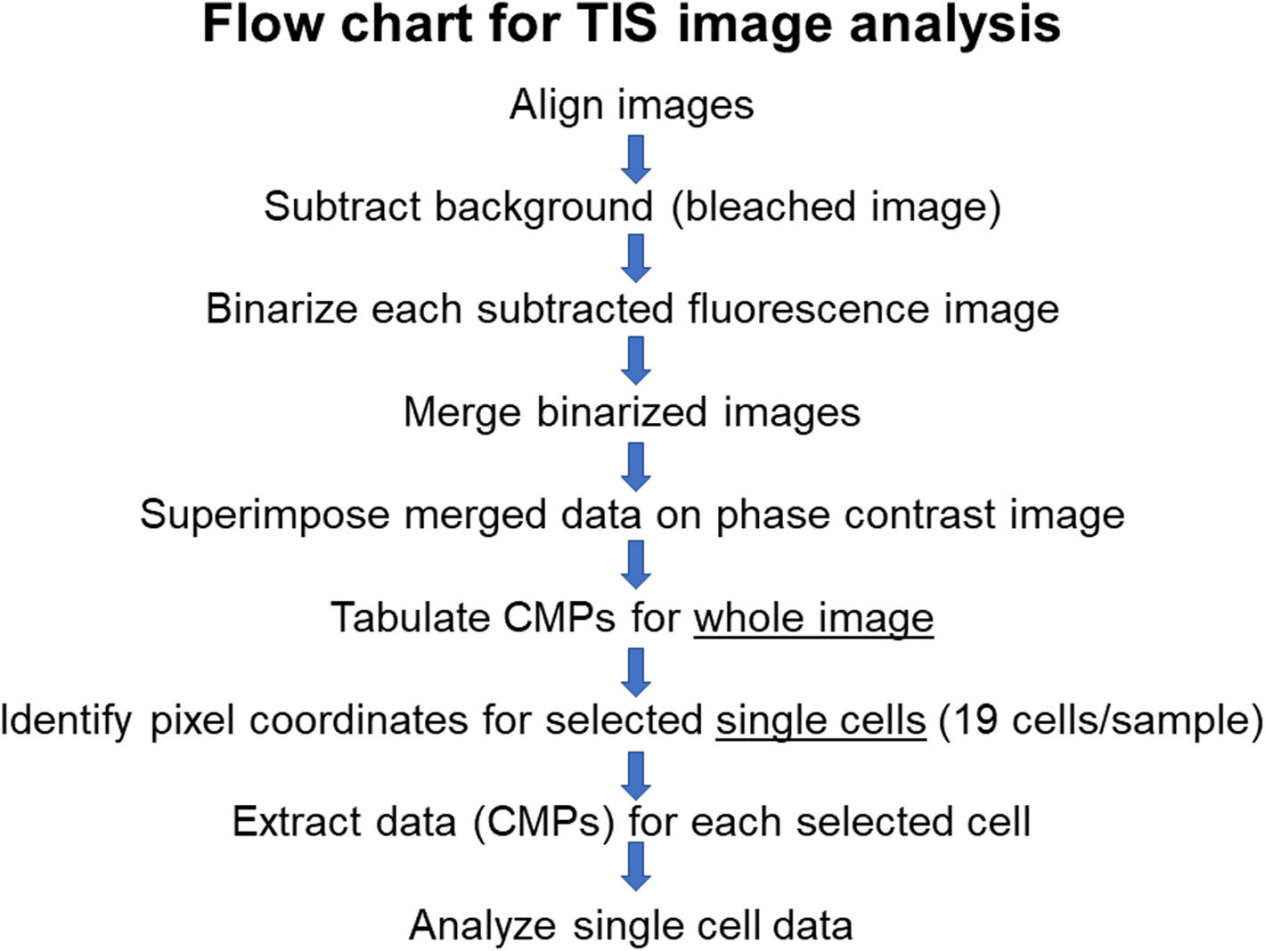
Flow Chart of TIS image analysis. The basic steps for image analysis for TIS are listed

The TIS system used was the TIS basic 4 (pi4 Robotics GmbH, Berlin, Germany). The system consists of a climate-controlled cabinet containing: a Zeiss AxioImager microscope with a Colibri.2 lighting system and a Plan-Apochromat 63X/1.0 Ph3 M27 water immersion objective; an SC4022M digital imaging system (Finger Lakes Instrumentation, LLC, Lima, NY); and a motorized pipette controlled by a robot. Software programs (developed by Reyk Hillert, Magdeburg, Germany) accompanying the TIS and used for data generation and analysis were: Image Registrator v.1.1 (for image alignment and background subtraction); Binary Center v.1.0.2 (for binarization of images); MoPPi v.1.1.3.8 (converts binarized .pgn files into a single .xml file); and MultiCompare v.0.9.0 (extracts CMP data from .xml files). A flow chart for TIS image analysis is shown in Fig. 2.

### Antibody calibration/optimization

All reagents (antibodies and phalloidon) were conjugated with fluorescein isothiocyanate (FITC) and obtained commercially (Table 2). FITC was the label of choice because it can be photo-bleached after imaging and prior to immunostaining with additional antibodies. In order to optimize antibodies for TIS we needed to calibrate the appropriate antibody dilution and exposure time for imaging the bound fluorescence. Using samples similar to those we used for our study, we tested each antibody at several different dilutions. We kept incubation time with each antibody constant at 30 min. The antibody concentration that resulted in a good fluorescence signal with minimal background was used and we experimented with exposure times of various durations to find the optimal exposure time for images to obtain good signals that were below saturation. After confirming concentration and exposure times, TIS runs were set up with the whole series of antibodies. The TIS procedure is summarized in a flow chart (Fig. 1). After imaging, bound FITC-conjugated reagents were photobleached. After the bleaching cycles the sample was re-imaged and the image used for background subtraction during subsequent image processing. The photo-bleached slide was then subjected to another round of immunostaining with the next marker. Table 2 lists the antibodies used, their gene names (where appropriate), Uniprot accession numbers, source of antibody, and catalog number of antibody.

**Table 2:**
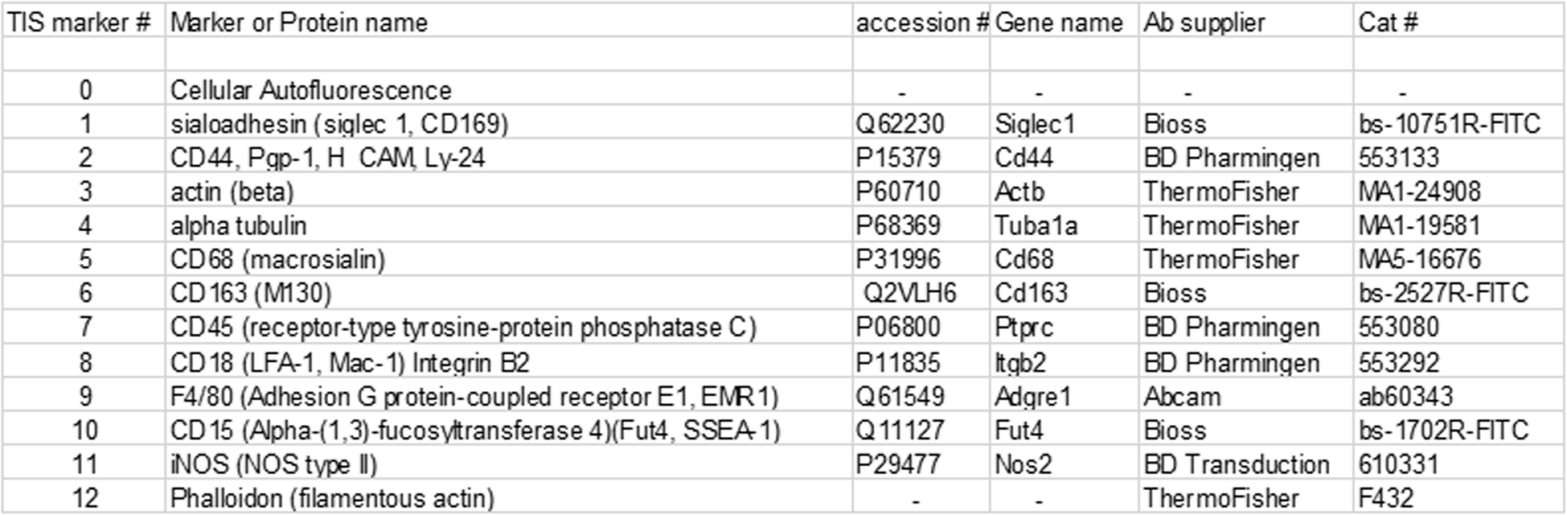
Basic information about the markers and the reagents used. Note that the numbering of the markers begins with 0 in compliance with the TIS software. The name of the marker and qualifiers or alternative names are provided in the second column, and accession numbers and gene names are listed in the third and fourth columns for markers that are single gene products (this excludes cellular autofluorescence and polymerized actin). Antibody suppliers and catalog numbers are in the fifth and six columns, respectively.

### Image processing for TIS

#### Whole image analysis

Following each run, images were subject to initial processing with the TIS software as outlined in Fig. 2. Images were first aligned to eliminate small shifts that may have occurred during the run. This step ensures that a given pixel is in the same position on all images. The shifted images were then subjected to background subtraction. These steps were done with the Image Registrator program. Whole images contained 2048 × 2048 pixels, although a 15-pixel margin around the periphery of each image was not included. In our TIS system with a 63X objective, a pixel in the captured image covers an area of 117 nm × 117 nm.

Our TIS runs contained a number of additional markers along with the 13 markers found in the final analysis. However, in order for us to compare the 6 samples in this study we needed to have good, artifact-free images for every marker in the runs for all 6 samples. In some cases, fluorescent debris, bubbles, or other artifacts prevented us from using an image and resulted in the omission of that marker from the final image collection.

The shifted, background-subtracted images for each marker were then reviewed to ensure that they were free of artifacts and were then subjected to binarization in the Binary Center program where a positive signal was either present (1) or absent (0). Threshold setting for binarization of the images from each marker was done manually and immunostained areas reaching the threshold were considered positive. All images used in this study were processed for binarization on the same day to ensure consistency.

Using the MoPPI program the binarized images for all 13 markers were merged into a .xml file which lists every pixel and the CMP present in that pixel. In this file each CMP is designated by a 13-character string of 1 s (when the protein is present) and 0 s (when the protein is absent) (i.e 1011001,000100).

**Fig. 3:**
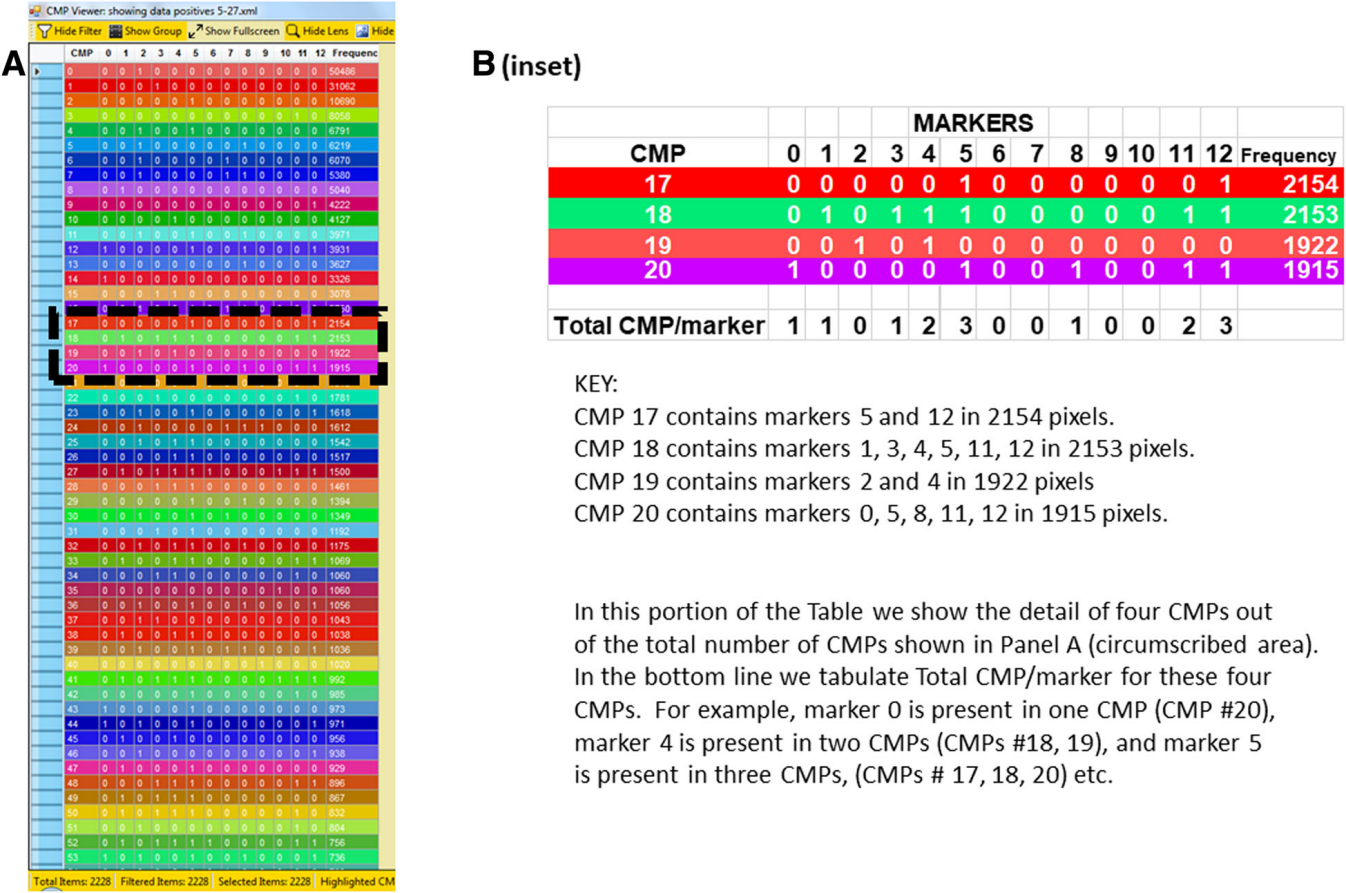
Panel **A**: A representative screenshot from the TIS software for the 5–27 sample. It shows the 54 most abundant CMPs in one of the merged, binarized images from this study generated by the MultiCompare program. This list shows the markers present [1] or absent (0), and the frequency (# of pixels; abundance) of each CMP in the full image. The number of different CMPs (2228) in the whole image is shown on the bottom line. An area (inset) defining four CMPs circumscribed with a dotted line is shown in detail in Panel **A**. The bottom line of the image (Panel **B**) shows the total CMP/marker for the CMPs in the example. See also Fig. 6

**Fig. 4:**
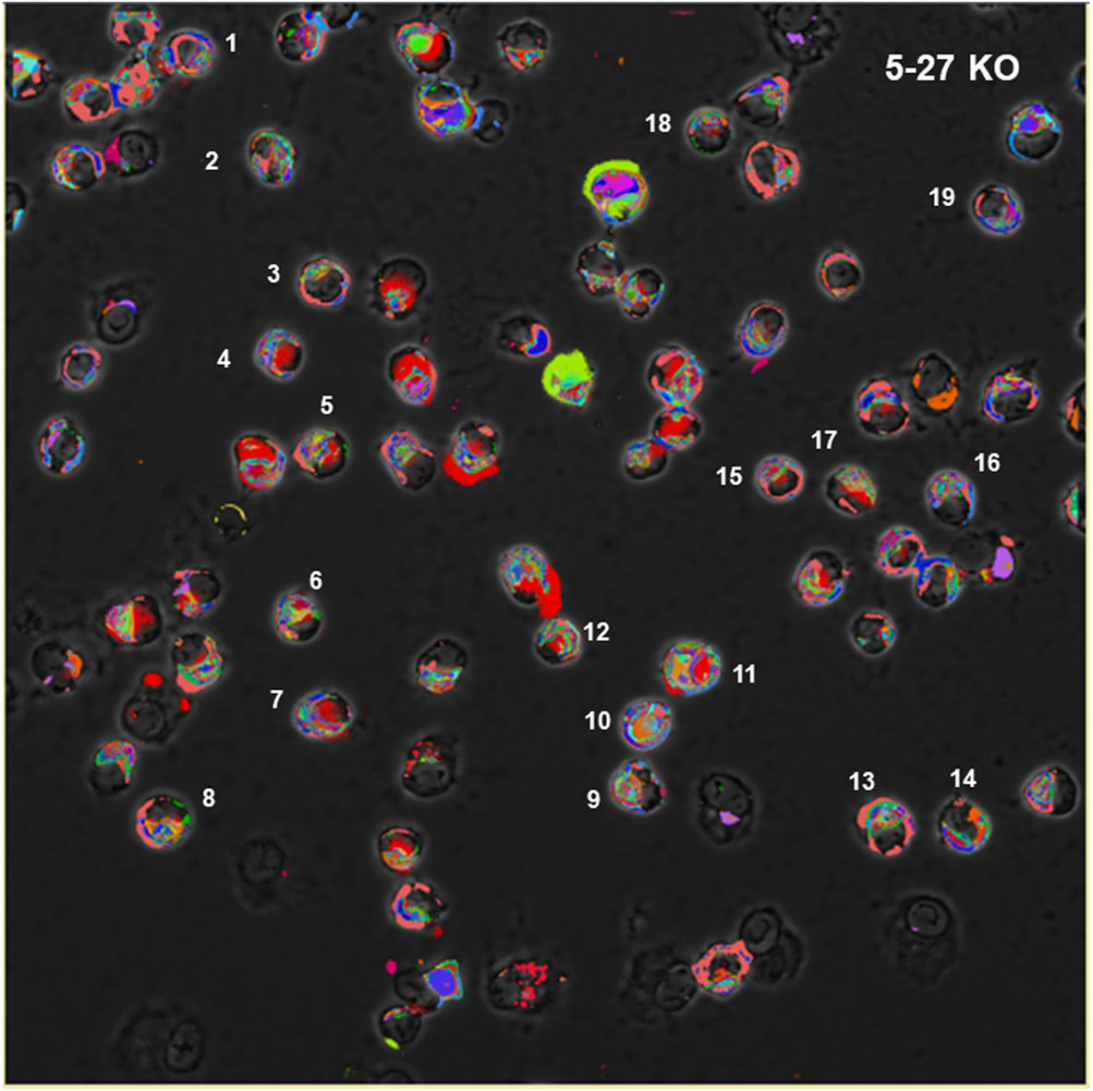
Selection of individual cells. In this image, a composite pseudocolored image has been generated from the binarized images of each of the fluorescent images from the 13 markers superimposed on a phase contrast image of the same cells. From each pseudocolored composite image 19 cells were selected and numbered. Cells that were chosen were separate from other cells, grossly normal in appearance, and away from the border of the image. Colors were automatically assigned by the TIS software as shown in Fig. 3

The .xml files were imported into MultiCompare to generate a table of all CMPs, each CMP was automatically assigned a color by the program, and their frequency (abundance) in the whole image was calculated (Fig. 3). The frequency is the number of pixels in an image containing a particular CMP. A screenshot from a representative image from a sample that is designated 5–27 (see top of figure) is shown (Fig. 3, Panel A) defining the 54 CMPs (out of 2228 CMPs) with the highest frequency. This 54 CMP table is only a portion of a table that included the 2228 unique CMPs present in the image of this sample and constitutes a graphic representation of the data in the .xml file for each image. A small part of the table in Panel A is circumscribed with a dotted line and shown in detail (Fig. 3, Panel B) using the same colors that were automatically assigned and shown in Panel A. CMPs are numbered (left hand column) in order of decreasing frequency (right hand column) and the presence or absence of each marker (labeled 0 to 12) is indicated in the intervening columns. Below the table we have added a row summarizing the data in this portion of the table by showing the total number of CMPs containing each marker. This information is superimposed on a corresponding phase contrast image and a pseudocolored image of the binarized data (Fig. 4) was generated using the CMP data and the assigned colors. Note that in some cases the intensity of the immunofluorescent staining was below the thresholds set during binarization. This resulted in some cells or parts of cells that were not pseudocolored. For additional analysis of the CMPs, the .xml files for each subject were converted to text files and read into SAS, Version 9.4.

#### Statistical analysis of whole images

Whole images from KO and SP-A1 groups were compared in several ways. In one analysis we compared a data set in which we determined the number of identical CMPs in the three KO samples and in the three SP-A1 samples, as well as the number of identical CMPs in two out of three members of each group (see Table 3). These totals were compared with an aligned rank test. This analysis focused on identical intact CMPs comprised of all 13 markers. In addition, we also compared groups by determining the similarity coefficients for each of 78 possible pairs of markers (i.e. marker #1 and marker #2, marker #1 and marker #12, etc). Furthermore, the full set of means of the 78 similarity coefficients were compared to assess the difference between the KO and SP-A1 groups. All of these analyses compared the overall similarity of the two experimental groups.

**Table 3:**
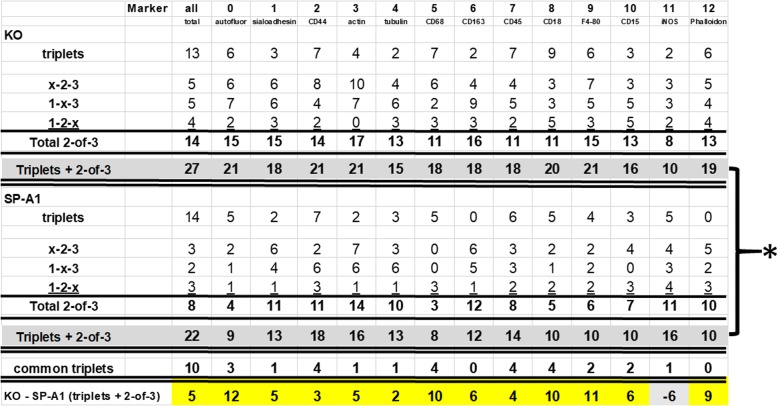
The 3 samples in each group, KO (top half) and SP-A1 (bottom half), were compared to identify CMPs present in all 3 samples (triplets) or in 2-of-3 samples

A total of 13 markers (0–12) were studied. The number of identical CMPs present within the 50 most abundant CMPs out of the 2228 total CMPs are listed in Fig. 3. Next a filter was used to select, from the total number of CMPs (i.e. 2228 for the sample shown), the 50 most abundant CMPs containing a particular marker. This was done for each of the 13 markers. Then, from the 50 most abundant CMPs for each marker, CMPs that were present in all three samples of each of the two groups (KO and SP-A1) were noted as “triplets.” Those present in 2 out of 3 samples were noted as “2-of-3” (x-2-3; 1-x-3, 1–2-x). For example, the designation x-2-3 denotes the absence of a CMP in sample 1 and its presence in samples 2 and 3). These numbers for the triplets and 2-of-3 are shown in columns labeled 0–12 for each marker. The total number of triplets, 2-of-3, and of triplets plus 2-of-3 samples are also given for the KO group and the SP-A1 group. These values were compared and found to be significantly different (*) using an aligned rank test (*p* = 0.0007). At the bottom of the table, the number of triplets that KO and SP-A1 cells have in common are listed, and below this there is a line giving the difference (KO – SP-A1) between the two groups (highlighted in yellow)

#### Single cell analysis

**Fig. 5:**
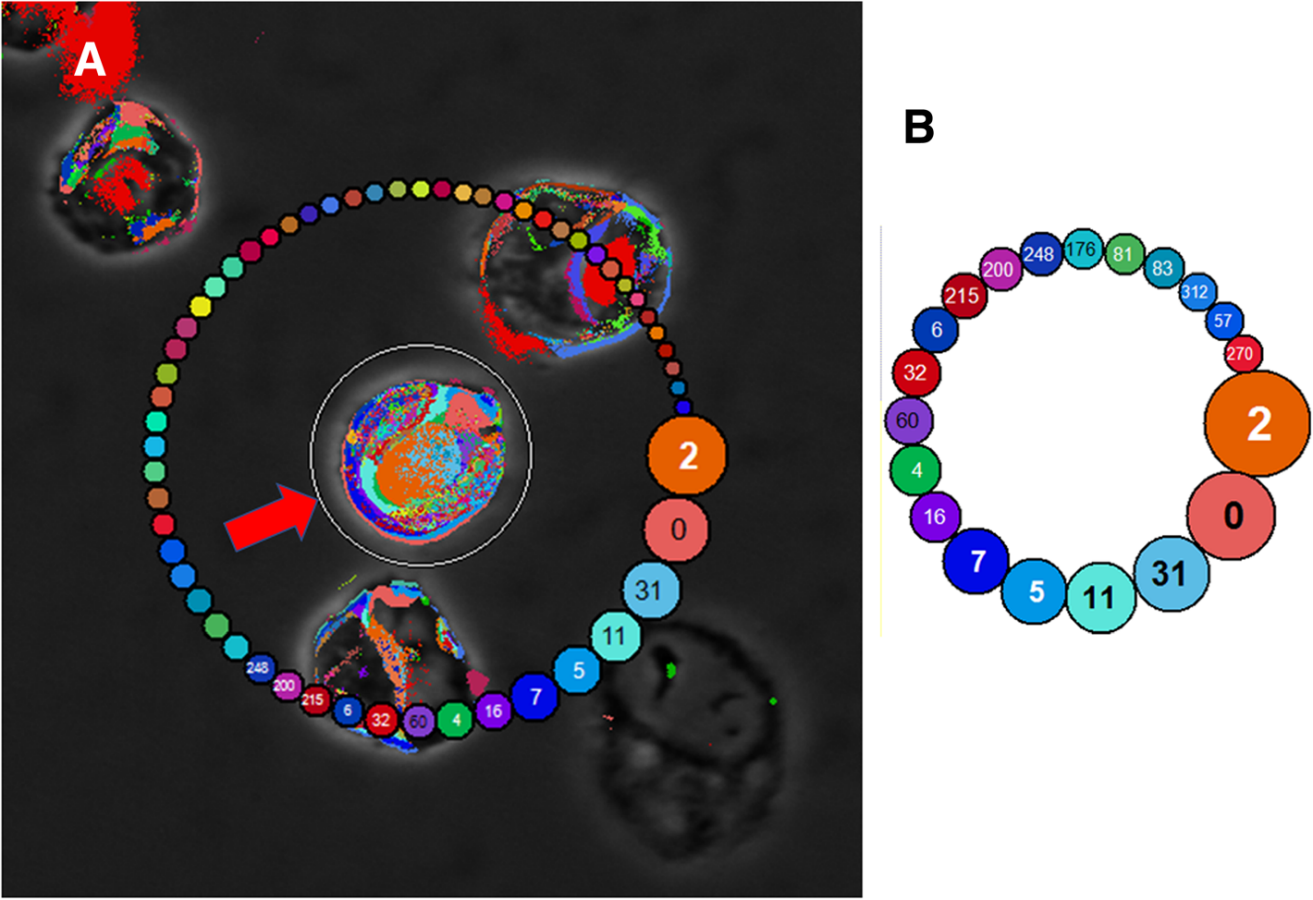
Analysis of individual cells. An example of this analysis using cell #10 from the 5–27 sample (see also Fig. 4). Each selected cell was circumscribed with a utility called a “lasso tool.” The inner ring (arrow) shows the area being analyzed. The outer ring is shown as a necklace or string of beads and depicts the CMPs present in order of abundance within the circumscribed area. The abundance of each CMP in the circumscribed cell is proportional to the size of the beads. The CMP numbers for some of the beads are not shown in the screen shot in Panel (**A**), but can be seen in Panel (**B**), which shows the top 20 CMPs for the circumscribed cell (arrow). The colors and CMP numbers are automatically assigned by the TIS software and correspond to the list of CMPs (based on frequency) for the entire image with all cells in that sample (see Figs. 3 and 4)

SAS data sets were also used to probe CMP data for single cells. This was done for 19 cells in each image (see Fig. 4). The selected single cells were grossly normal in appearance and did not touch any other cell. Using the MultiCompare program, a utility called a “lasso tool” makes a circle around each selected cell (Fig. 5, Panel A; inner ring, see arrow) which generates a necklace plot (outer ring) in which the CMPs present are shown in order of decreasing frequency. The size of each bead in the necklace plot is proportional to its frequency; the size of the necklace plot was then adjusted to show the 20 most abundant CMPs within the selected cell (Fig. 5, Panel B). The colors and numbers correspond to the data shown in Fig. 3, Panel A. The CMPs were recorded and converted to the corresponding 13-character signature (markers present = 1 or absent = 0) for each CMP shown in Fig. 3, and for each of the total 114 cells analyzed. For analysis of single cells the pixel coordinates for each of the selected cells were initially determined using Image J software (https://imagej.nih.gov/ij/download.html) and then converted to be compatible with the data in the SAS file of the whole image data. These coordinates were then used to select the pixels comprising each cell and to determine the CMPs present in those pixels. The single cell data extracted from the SAS files and the data generated by the “lasso” tool were compared to confirm that they were identical.

## Results

### Whole image analysis

Whole image analysis includes all of the cells in the entire visual field with a 63X objective comprising 2048 by 2048 pixels. In this study we built on our published proteomics studies [21, 22, 26], where we have shown that treatment of SP-A KO AM with exogenous SP-A1 resulted in significant differences in the AM proteome. Here we demonstrate changes in the AM toponome under similar conditions. For the current study we used an experimental animal protocol identical to one we have used in studies of the AM proteome [26].

#### Markers

The markers used in the present study were selected from a larger collection of antibodies. The 13 markers chosen for further study (Table 2) produced the most reliable, artifact-free signals. Several markers were eliminated from the final analysis because we were unable to obtain high quality, artifact-free images for all 6 samples. One of the markers we used was the autofluorescence (AF) of the AM at the beginning of the TIS run. AF, which has been shown to be heterogeneous, has been previously used as a useful characteristic in myeloid cell analysis [49]. Given the intracytoplasmic organelle localization of AF, potential sources for the AF include NAD(P)H, flavins, ceroid/lipofuscin, bilirubin, porphyrins, among others [49]. In the present study most of the AF was punctate or granular in nature (see Additional file 1: Figure S1), and possibly related to the bactericidal capacity of the cells, a function potentially shared with another marker (iNOS). It should be noted that this AF was completely eliminated by the standard series of photobleaching cycles. Several of the markers may play roles in endocytosis or phagocytosis (sialoadhesin, CD44, CD68, CD163, CD15) and many of the markers are likely to be involved in cell-cell and cell-matrix interactions (sialoadhesin, CD44, CD68, CD45, CD18, F4/80, and CD15). The effectiveness of these AM functions depends on the motility of the AM and several markers specifically relate to that ability (actin, tubulin, phalloidon).

#### Analysis of data from whole images

Initial processing (Fig. 2) was done with whole images containing all of the cells in the 63X field of view. Because the number of cells in each image varied, our analysis is qualitative rather than quantitative. The MultiCompare program generated a table (Fig. 3, Panel A) listing every CMP in order of frequency/abundance (column on left side), assigning colors, noting the presence or absence of each individual marker in columns labeled 0–12, and giving the frequency (abundance; # of pixels) of each CMP in the column on the right. This program also generated a pseudocolored image (Fig. 4) of the cells with CMP colors that corresponded to those in the list (Figs. 3 and 5).

**Fig. 6:**
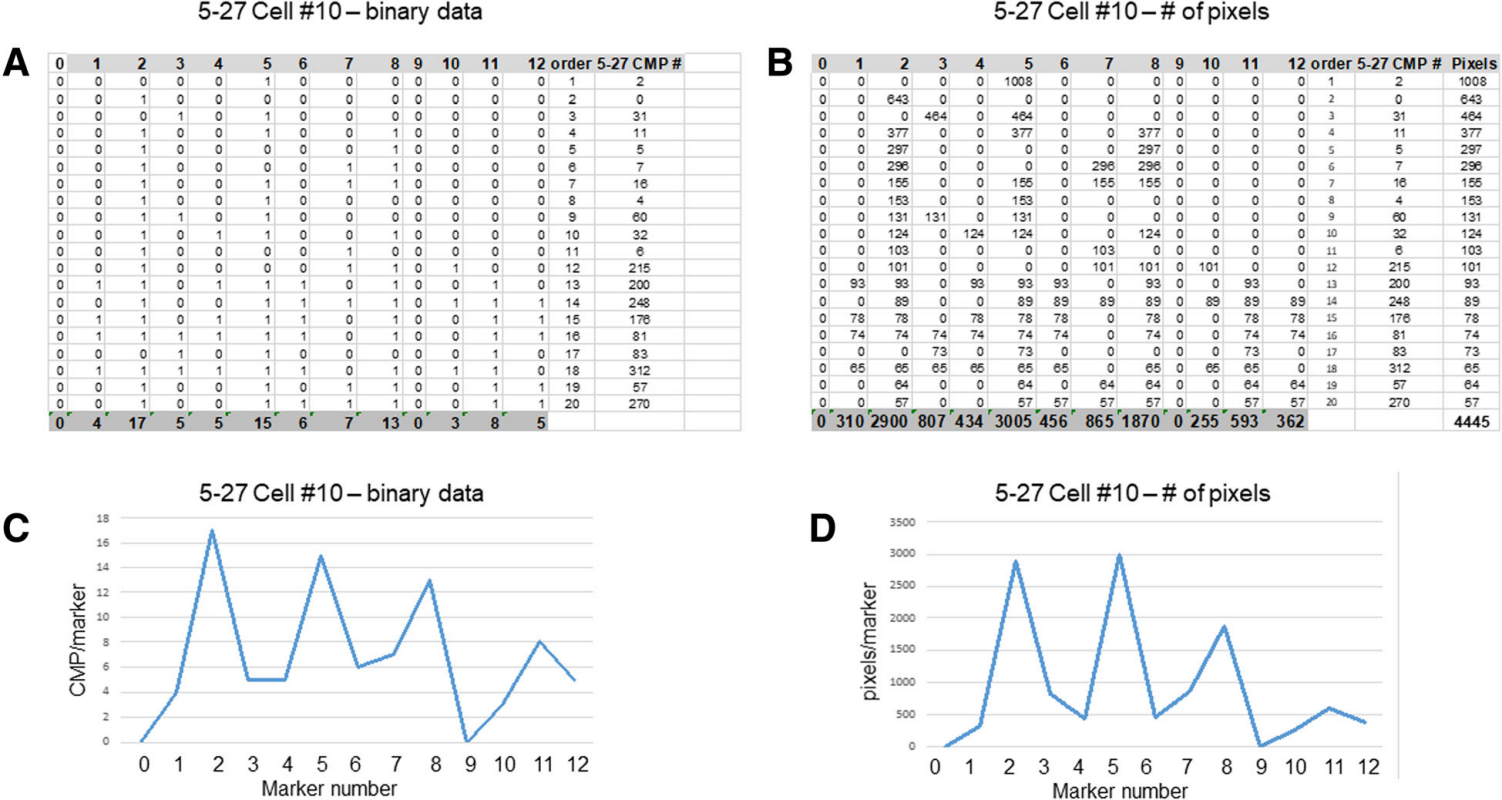
Representative CMP summary. Panel (**A**) gives a binary representation (present = 1; absent = 0) of the CMP composition of each of the 20 most abundant CMPs for a representative cell (Cell #10 from the 5–27 sample; see also Figs. 4 and 5). Columns 0–12 are for each of the 13 markers analyzed. The bottom line in the table shows the total number of CMPs (out of the top 20) containing each of the 13 markers (as in Fig. 3, Panel **B**. For example, the total number of CMPs containing marker 1 is four. The column labeled “order” shows the order of abundance [1–20] for the top 20 CMPs. The column marked “5-27 CMP #” gives the CMP number derived from the full image (Fig. 4) of the 5–27 sample (see Fig. 3, Panel **A**. Fig. 6, Panel (**B**) is organized as in Panel (**A**) and consists of data from the same cell used for Panel A except that it shows the number of pixels. In Panel (**B**) the number of pixels (last column) occupied by each CMP is recorded for each marker present in the top 20 CMPs. The total number of pixels occupied by each marker is given in the bottom line of Panel **B**. For example, the total number of pixels occupied by CMPs containing marker 2 is 2900. The columns labeled order and CMP# are as described with Panel (**A**) (above). Panel (**C**) provides a graphic “signature” of the binarized data in Panel (**A**), summed up in the bottom line of Panel (**A**) (highlighted gray and bold). Panel (**D**) depicts the summed data from Panel (**B**) (bottom line). The x-axis indicates the marker number (0–12). The y-axis in Panel (**C**) shows the number of CMPs containing each marker, and in Panel (**D**), the number of pixels occupied by each marker

**Fig. 7:**
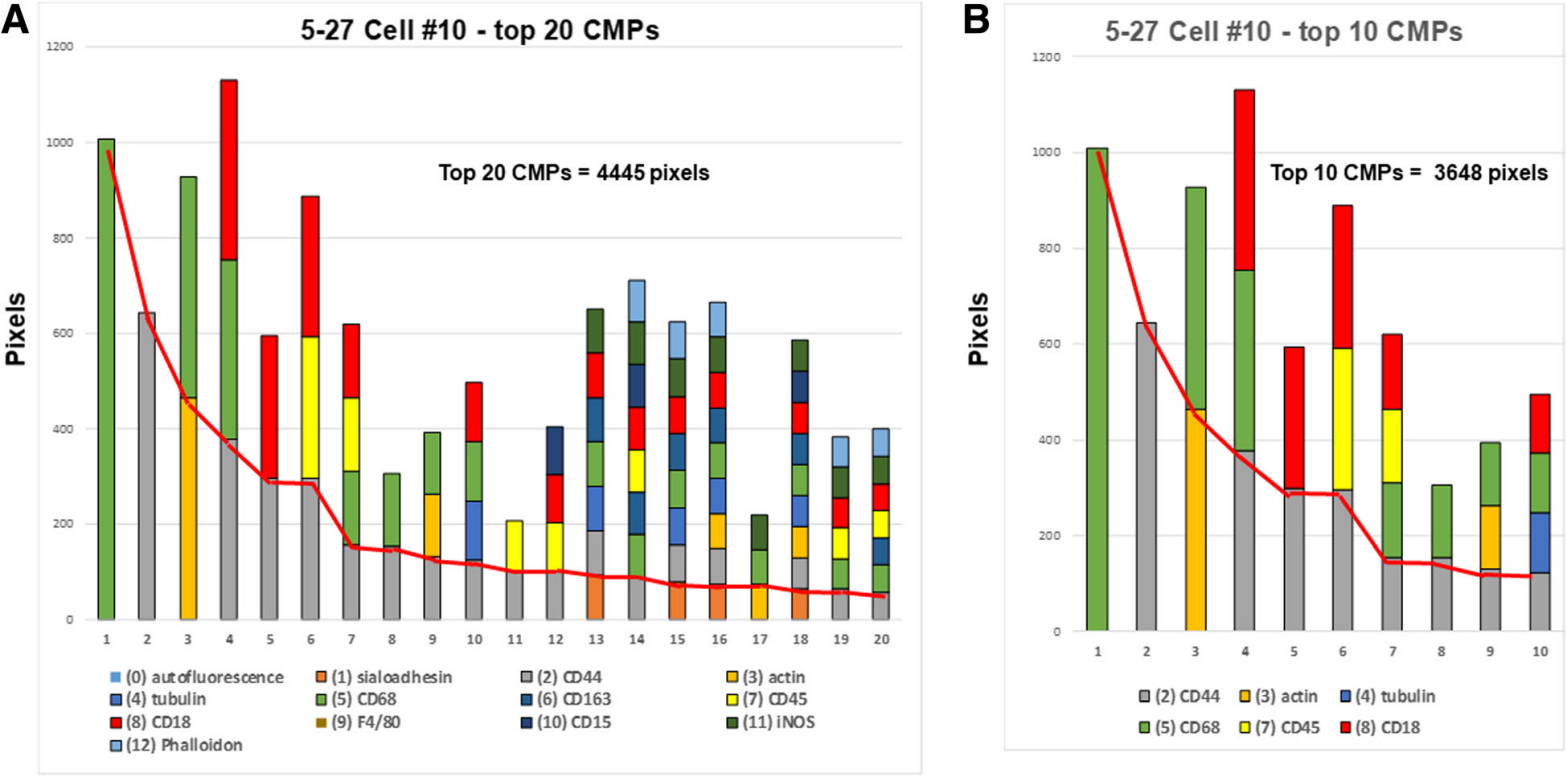
Segmented bar depiction of CMP composition. The solid red lines in both Panels show the number of pixels/CMP. In Panel (**A**) segmented bars depict the marker constituents of the 20 most abundant CMPs for the same cell characterized in Fig. 6. Each segment of the bar represents the marker present in that CMP. The number of segments depict the number of markers present in the particular CMP. The key for the color coding of the markers is shown below the graph. The height of each segment below the solid line corresponds to the number of pixels occupied by that CMP. The total number of pixels (4445) occupied by the top 20 CMPS is given. Panel (**B**) shows the bars for the 10 most abundant CMPs, the markers present in each CMP, and the number of pixels (below the solid red line) occupied by each CMP, as well as the total number of pixels (3648) occupied by the top 10 CMPs. Note that: a) the top 10 CMPs constitute 82% (3648 pixels) of the total pixels (4445 pixels) occupied by the top 20 CMPs. **B**) As one moves from the most abundant to the less abundant CMPs the diversity of the CMP (i.e. the number of markers contributing to the particular CMP) increases

The table shown in Fig. 3 Panel A shows a screenshot that lists the 54 most abundant CMPs in the entire image of the 5–27 sample. Images of other samples (not shown) contained varying numbers of cells and CMPs (average of all samples = 2192 CMPs; range 1739–2616). Approximately 20% of the total CMPs in each sample were found in 50 or more pixels in the whole image comprised of 2048 × 2048 pixels. In this example (from the sample designated 5–27), there are a total of 2228 distinct CMPs (see bottom line of Fig. 3, Panel A). Panel B extracts a portion of this table to highlight the detail of 4 CMPs. In reviewing the images (Fig. 4) it was immediately obvious that the macrophages constituted a heterogeneous cell population, varying not only from sample to sample, but also within cells of the same sample. Figs. 3, 4, 5, 6, 7 all depict data from the sample designated 5–27.

We attempted to characterize this heterogeneity by focusing on the more abundant CMPs. We initially examined the 50 most abundant CMPs from each image (Table 3) and compared the 3 subjects from each experimental group (KO and SP-A1) to each other.

### CMP presence in samples under study

#### a) Triplets

Our first step was to determine which CMPs were present within the 50 most abundant CMPs in all 3 samples of each experimental group which we referred to as “triplets” (see Table 1). We found that in the KO samples 13 of the top 50 CMPs were present in all 3 samples and in the SP-A1 samples there were 14 of 50. Of these, 10 CMPs were present in all 6 samples (i.e. in cells of KO and SP-A1-treated mice) (Table 3; common triplets).

Next, we used a function of the software that allows us to select only the CMPs that contain a given protein (Table 3). For example, when marker 1 (sialoadhesin) was selected and the filter applied, we obtained a list of the top 50 CMPs that contained marker 1. The column below marker 1 in Table 3 shows that in KO mice three CMPs were present in all three members of the group (triplets) among the most abundant CMPs, but only in two members of the SP-A1 group. We did this for each of the 13 markers used for the study and the results are listed in Table 3. In all cases (except tubulin and iNOS) the number of “triplets” for each marker in the SP-A1 group is lower than or equal to that in the KO group. This observation seems to indicate more consistency (or less heterogeneity) among the 3 subjects in the KO mice as compared to the three SP-A1 mice.

#### b) Two-of-three

We then did a similar analysis looking at the number of identical CMPs (among the 50 most abundant CMPs containing each marker) in each combination of two out of three individuals (i.e. x-2-3; 1-x-3; 1–2-x). The number of common CMPs in the comparisons of two-out-of-three individuals is listed (Table 3), followed by the number of common CMPs in both the triplets and the 2-of-3 comparisons. It is immediately obvious that there are more instances where 2-of-3 matches occur in the KO mice than in the SP-A1 mice. In all cases, except with iNOS, there are more matches for each marker among samples in the KO group than the SP-A1 group. In several cases there are more than twice as many 2-of-3 matches in the KO group vs the SP-A1 group (autofluorescence, (15 vs 4); CD68, (11 vs 3); CD18, (11 vs 5); and F4/80, (15 vs 6)).

The bottom line in Table 3 further emphasizes the differences between KO and SP-A1 by showing how many CMPs (triplets and 2-of-3) are consistently present in KO. These data were compared using an aligned rank test and found to be significantly different (*p* = 0.0007). These observations further strengthen the idea than the KO group is more uniform than the SP-A1 group. In other words, the three KO individuals were more like each other than the three SP-A1 individuals.

The above statistical comparison was a fairly stringent one because it compared the presence or absence of CMPs in their entirety (all 13 markers). However, during our analysis we frequently observed groups of CMPs that were highly similar to one another. For example, CMPs that are identical with respect to eight markers but vary for the other five markers could include 2^5^ or 32 similar CMPs that would not have been included in the above comparison. In order to assess the impact of some of this variability, we constructed an analysis of similarity coefficients in which all 78 possible pairs of markers (i.e. marker #1 and marker #2; marker #4 and marker #12, etc) were compared between KO and SP-A1 cells. When this was done 50 of the 78 similarity coefficients were significantly different (*p* < 0.05) between groups. Furthermore, when the means of the 78 similarity coefficients were compared between KO and SP-A1 groups the analysis found that the KO and SP-A1 groups were highly significantly different (*p* < 0.0001).

### Analysis of single cells

Because of the heterogeneity of the AM that is described above, we chose to analyze single cells in addition to the whole image analysis already described (Fig. 2). We selected a total of 114 cells (57 cells for each group – KO and SP-A1–19 cells from each of six subjects (3 KO and 3 SP-A1)). The selected cells fulfilled the following criteria: they were free standing (not clumped or overlapping), totally within the analyzable area of the image (not in margins of image), and appeared to have grossly normal morphology. An example showing the selected cells is depicted in Fig. 4. We hoped that with this large number of cells and the selection criteria we used, any potential selection bias, if not totally eliminated, would be minimized significantly. The pseudocoloring of this image corresponds to the colors in the list of CMPs for this sample (Fig. 3) and are automatically assigned by the program. Cells contained an average of 4814 pixels (range 2072–8222) and each cell had between 38 and 463 CMPs. We focused on the 20 most abundant CMPs in each cell.

### CMPs and pixels in single cells

Two tables were generated for each of the 20 most abundant CMPs for each cell. A representative pair of these tables for one cell (Cell #10, also depicted in Fig. 5) from the 5–27 sample is shown in Fig. 6. The first table consists of the binary data (present = 1 or absent = 0) for each CMP (Fig. 6, Panel A) and the second table contains abundance (number of pixels) for each CMP (Fig. 6, Panel B). Each of the 20 CMPs is defined in the rows of the table. The tables contain columns labeled 0–12 denoting each of the 13 markers (see Table 2). The next column in both tables (Panels A and B) shows the order [1–20] of the top 20 CMPs from most abundant to least abundant. Then this is followed (Panels A and B) by a column giving the CMP number (5–27 CMP#; from data in Fig. 3) for each of the top 20 CMPs in that cell. The number under 5–27 CMPs corresponds to the relative abundance of each CMP based on the composite image generated from the binarized images with all 13 markers (see Fig. 3). For example, in Fig. 6, Panel A under “5-27 CMP #”, #2 is the most abundant CMP for this cell (#1 under “order”), but the third most abundant CMP in the image containing all of the cells in the 5/27 sample as shown in Fig. 3 (note that the most abundant CMP in Fig. 3 is numbered 0). Similarly, the tenth most abundant CMP (#10 under “order”; #32 under “5–27 CMP #”; Fig. 6A) is the 33rd most abundant CMP for the entire image of the 5/27 sample (Fig. 3 and Fig. 6A; but is labeled #32 due to #0 being the first CMP). The image from the 5–27 sample had a total of 2228 CMPs (see bottom line, Fig. 3, Panel A). The 2228 CMPs represent the collective number of all of the CMPs in all of the cells in the 5/27 sample. In Fig. 6, Panel B the final column lists the number of pixels occupied by each of the top 20 CMPs and gives a grand total of 4445 pixels for this cell. The bottom line of each table gives the total number of CMPs containing each marker for a given cell (Panel A) and the total number of pixels occupied by each marker in the top 20 CMPs for the same cell (Panel B).

**Fig. 8:**
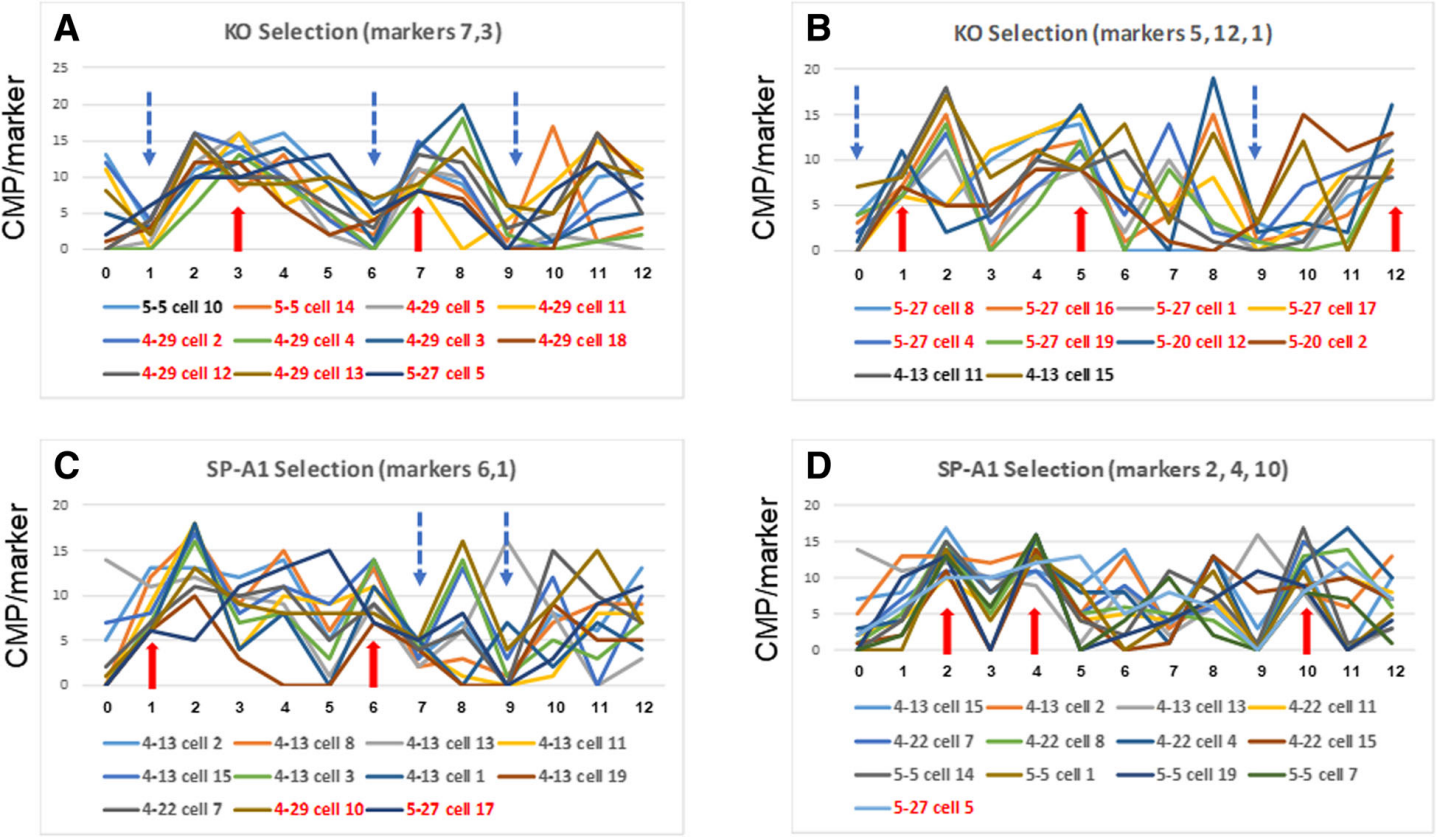
CMP signatures selected by high levels of markers that distinguish between groups of KO and SP-A1 cells. Examples of these are shown in Panels **A**-**D**. In this Figure we selected cells by screening for high levels of particular markers at or above the limits listed in Table 4. The markers being used for the selection are indicated by the red arrows  in each panel. The CMP summaries for each cell (see Fig. 6, Panel (**A**), bottom line) were screened to determine whether there were high levels of each marker (i.e. a marker that was present in a large number of the top 20 CMPs). Using the limits shown in Table 4, the cells with high levels of each marker were identified. In the key below the graphs, cells in the KO group are shown in red and SP-A1 cells are in black. Panel **A** depicts cells selected with markers 7 and 3 (9 of the 11 cells selected are in the KO group). Panel **B** shows cells selected with markers 5, 12, and 1 (8 of 10 are KO cells). Panel **C** depicts cells selected by markers 6 and 1 (9 of 11 are SP-A1 cells). Panel **D** shows cells selected by markers 2, 4, and 10 (12 of 13 are SP-A1 cells). Features (high or low levels of a given marker) that were not used in the selection, but are present in most, or all, members of a subgroup and may be useful for characterizing that subgroup are indicated with a dashed blue arrow

**Fig. 9:**
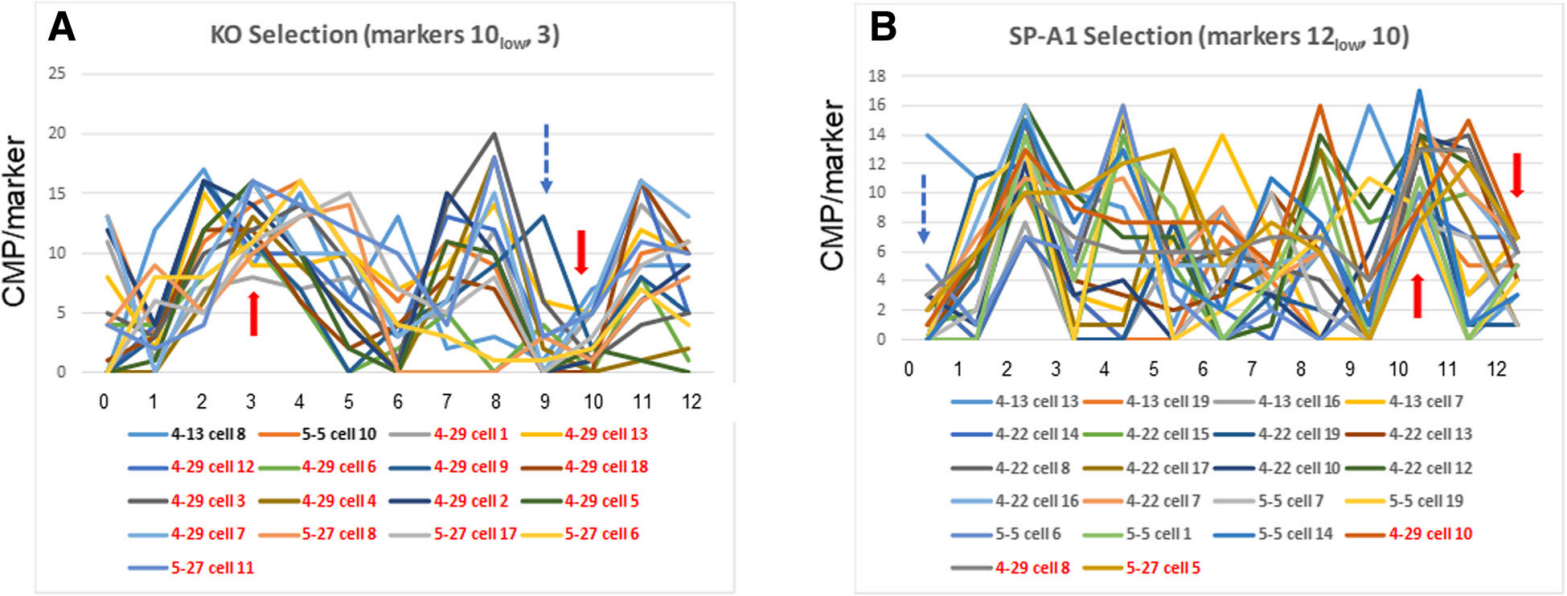
Selection of subgroups with low levels of a marker. This Figure shows two examples where groups consisting of mostly KO or SP-A1 cells were selected by first applying a screening step in which cells were selected by levels below the limits listed in Table 4 (i.e. low levels). A second screening step selected cells from the first screen that were at or above the limit (from Table 4) for the given marker. Arrows are used as in Fig. 8. Panel **A** shows cells selected first by low levels of marker 10, then with high levels of marker 3 (15 of 17 are KO cells). Panel **B** shows cells selected by low levels of marker 12 and then with high levels of marker 10 (19 of 22 are SP-A1 cells). In the legend of the graph KO cells are in red and SP-A1 cells in black

Next, we generated a line graph from each of the totals (bottom line of each table in Panels A and B) providing a “signature” or “snapshot” of the makeup of each cell (Fig. 6, Panels C and D). These depict either the total number of CMPs (out of the top 20 CMPs) containing each marker (Panel C) or the total number of pixels containing each marker (Panel D). Although there are differences between the two graphs, the general pattern of peaks (i.e. presence of many CMPs with a given marker) and valleys (i.e. only a few CMPs with a given marker) is very similar in both cases. Line graph “signatures” like those shown in Fig. 6, Panel C, are also used in Figs. 8 and 9. If we examine Fig. 6, Panel C, we can see that there are peaks for markers 2, 5, and 8, meaning that in this cell many of the top 20 CMPs contain markers 2, 5, and 8 (see bottom line of Fig. 6A). We can also see that none of the top 20 CMPs contain markers 0 and 9. As a result one sees, in terms of CMP/marker, 0 at markers 0 and 9 These plots served as a summary of the marker content or CMP signature of the 20 top CMPs for each cell and allowed us to identify groups of cells with similar characteristics, even though their CMPs were not identical. Although this figure represents a single cell, similar plots were made for the 114 cells comprising this study. With all of the cells, the plots graphing the binary data and those graphing the number of pixels were very similar, as shown in this example.

### Marker content in top CMPs in single cells

A series of segmented bar graphs (Fig. 7) with each segment representing one marker, illustrate the marker content of the top 20 CMPs in the same cell as characterized and depicted in Figs. 5 and 6. The red solid line crossing the bar graph shows the number of pixels for each CMP (taken from Fig. 6, Panel B) and shows that as one moves from the most abundant CMPs to the less abundant CMPs, the number of pixels is decreasing, and in many cases the number of markers in each CMP is increasing. The y-axis represents the number of pixels occupied by each CMP as shown by the red line, as well as the number of pixels for each marker (each segment) in each CMP in the bar graph. The x-axis denotes the top 20 [1–20] or the top 10 [1–10] CMPs. For example, CMP #6 in Fig. 6A (the 6th most abundant CMP in the cell) consists of three markers (see also Table 2 for marker numbers and names: markers 2 (CD44), 7 (CD45), and 8 (CD18)). As shown in Fig. 6 Panel B, this CMP occupies 296 pixels out of a total of 4445 pixels occupied by the top 20 CMPs. CMP #6 consists of three components and each segment on the bar graph for CMP #6 has 296 pixels giving the 3-component bar an apparent total value of 888 pixels. However, because the three markers are in the same CMP, they occupy only 296 pixels within the image of the cell, as depicted by the red line.

The graph for the top 20 CMPs shown in Fig. 7 Panel A, provided a considerable amount of information. **First**, the top 20 CMPs contained all 13 markers. **Second**, there was much more diversity (more markers/CMP) in the less abundant CMPs. **Third**, the top 10 CMPs (Panel B) constituted 82% of the total pixels occupied by the top 20 CMPs. These three trends were consistent in all 114 of the cells analyzed. For example, in the 19 cells analyzed from the 5/27 sample, the top 10 CMPs occupied an average of 71% (range 66–85%) of the pixels covered by the top 20 CMPs.

It was evident that considering the top 20 CMPs resulted in an apparent over-representation of pixels for CMPs with multiple components. If we restricted our analysis to the top 10 CMPs (Panel B), a total of 6 markers are present. This means that the high degree of diversity shown by the presence of all 13 markers was actually due primarily to the contribution of the less abundant CMPs (11th–20th).

These graphs were used as a qualitative tool, rather than a quantitative tool, so absolute values were not of particular concern. This graphic representation gave us a means to scan for conserved or unique CMP patterns that could represent a number of cells with a common phenotype. We used these graphs to identify conserved patterns or phenotypes among our subjects.

### Comparison of single cells between SP-A1 and KO

In an attempt to find ways to compare KO and SP-A1 cells we probed a file composed of the CMP summaries (see Fig. 6, Panel A, bottom line) for all 114 cells in the study. Our initial inquiry of these data was done as described in Table 4. On the left, the marker numbers are listed (0–12) and names, followed by a column with the **maximum** value for the number of CMPs (out of the top 20 CMPs) containing that marker in at least one cell out of the 114 cells analyzed. For example, the value of 14 for marker 0 indicates that in at least one of the 114 cells, marker 0 was found in 14 of the top 20 CMPs. We then set a limit of about one half of the maximum value (rounding down in the case of odd numbers (i.e. for a 13 Max value we set a limit of ≥6; 15 was ≥7, etc). This limit is basically a threshold that defines high and low levels of a given marker in the top 20 CMPs. This method is roughly analogous to the gating done in flow cytometry to define “hi” or “lo” levels of a given marker.

**Table 4:**
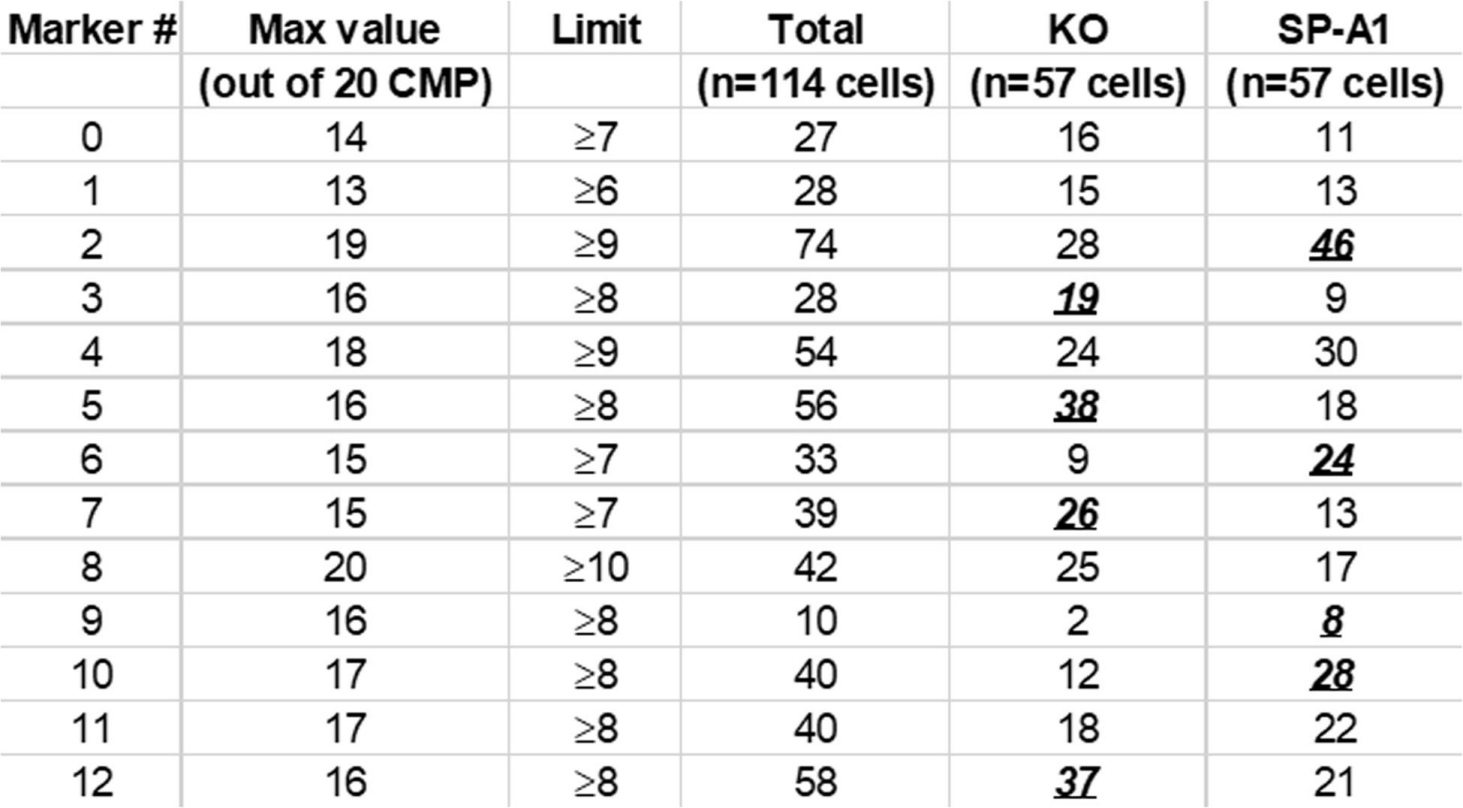
Summary table for high levels of the 20 most abundant CMPs in the total cell population (*n* = 114) analyzed

Marker # and name are listed in the two columns on the left (additional name details are in Table 2. For each marker (left hand column) the maximum number of CMPs out of the 20 most abundant CMPs (Max value) present in at least one cell is shown. For example, a value of 14 for marker 0 means that in at least one cell of the total 114 cells there were 14 CMPs among the 20 most abundant CMPs containing marker 0, and that none of the 114 cells analyzed contained more than 14 CMPs with marker 0. For marker 8, on the other hand, there was at least one cell out of the 114 analyzed that contained marker 8 in all 20 top CMPs, hence Max value for marker 8 is 20 (the highest that it can be). Next, a “limit” or threshold was set of about one half of that maximum and then we selected the cells out of the 114 cells that expressed the highest numbers (at or above the limit) of CMPs containing each marker. The resulting cells were then categorized as being either in the KO or SP-A1 experimental groups. In the case of marker 0, a total of 27 cells (out of 114) were at or above the limit of 7. Of the 27 cells, 16 were among the 57 analyzed cells in the KO group and 11 were in the SP-A1 group. Markers that were heavily represented in one group and of potential use as a selection tool are in bold, italicized, underlined type

Successive columns in Table 4 show the total number of cells out of the 114 cells analyzed that were at or above the threshold limit. For example, out of the 27 cells that had high levels (at or above the limit) for marker 0, sixteen were found in KO samples and eleven in the SP-A1 samples. Marker 6, is found above the threshold limit in 33 cells, but 24 of these cells are from the SP-A1 group, indicating that it could be a useful marker to distinguish between the 2 groups. Table 4 shows that CMPs with some markers are much more abundant in the KO cells (markers 3, 5, 7, and 12) and some other markers (markers 2, 6, 9, and 10) are much more abundant in the SP-A1 cells and these are in bold, italicized, underlined print. In some cases the differences of cells containing high levels of CMPs for a given marker between groups (KO vs SP-A1) are small as depicted (marker 1: 15 vs 13 and marker 11: 18 vs 22) and probably indicate that these markers would not be useful in discriminating between groups.

Note that although Table 4 shows the cell numbers at or above the limit shown in the third column, the data below the limit (which is not given in Table 4) are equally informative. For example, in the case of marker 10 (as shown in Table 4), there are forty cells (i.e the sum of the last two columns) out of the 114 total cells that are < 8 (vs seventy-four that were < 8 and forty-five of these are KO cells and twenty-nine are SP-A1 cells; not shown). Therefore, selection criteria for a given marker could be the cells at or above the limit (≥8), or it could be the cells below the limit. This information provided the basis for our initial categorization of the two experimental groups and denote characteristics of cells that may be useful in defining phenotypes/subgroups dependent on SP-A1 or on the absence of SP-A.

### KO vs. SP-A1

We used several sequential rounds of the screening method described above to identify cells that were highly enriched in one group versus the other. The screening for all 114 cells involved the data used to generate line graphs such as those shown in Fig. 6C and D. These graphs were a useful screening tool because they provide a graphic representation summarizing the most abundant CMPs in each cell. The line graph “signatures” (each in a different color) for the cells meeting these criteria are shown in Fig. 8A. These graphs (see Fig. 6, Panel C) plot the number of CMPs (out of the top 20 CMPs) that contain a given marker on the y-axis and the marker number on the x-axis. The cell information (sample name and cell number; eg. 5–27 cell #10) and the color key for the lines are listed below the graph.

The features responsible for each selection are indicated by the large red arrows. For example, in Fig. 8A, the selection was done with markers 7 and 3. We first screened for all cells with higher levels of marker 7 (CD45), based on the limit given in Table 4. We then subjected the results of that screening to a second selection in which we only retained the subset that also had higher levels of marker 3 (actin). This strategy resulted in a total of 11 cells, 9 of which were from the KO group (in the red print) and 2 were from the SP-A1 group (shown in black print). Fig. 8A shows the line graphs for all of the cells meeting the selection criteria and although they differ in many respects, they all show the relatively high values for markers 3 and 7. In some cases when the line graphs are shown together several other features present in most or all of the selected cells become evident that are also useful in defining this phenotype/subgroup of cells. These are indicated with blue dotted line arrows and include low levels (below the limit in Table 4) of marker 1 (sialoadhesin), marker 6 (CD163), and marker 9 (F4–80). The result is a description for this subgroup/phenotype that includes relative amounts of 5 markers (i.e. CD45^hi^, actin^hi^, sialoadhesin^lo^, CD163^lo^, and F4–80^lo^).

Several other examples are shown in Figs. 8 and 9. In Panel B a similar sequential selection using markers 5 (CD68), 12 (phalloidon), and 1 (sialoadhesin) identified 10 cells of which 8 belonged to the KO group. As in panel A, with the grouped line graphs using the initial selection criteria, we were also able to see that this subgroup had low levels of marker 9 (F4–80) and all of the cells except one SP-A1 cell (4–13 cell 15) had low levels of marker 0 (autofluorescence). Considering these five markers (i.e. CD68^hi^, phalloidon^hi^, sialoadhesin^hi^, F4–80^lo^, and autofluorescence^lo^) we had a subgroup of 9 cells, of which 8 were KO cells. Note that although both of these examples (Fig. 8A and B) select primarily KO cells and that in one group sialoadhesin (marker #1) is high, and in the other it is low.

Panels 8C and 8D show two other selection strategies that resulted in enriched populations of SP-A1 cells. Panel C shows the selection (from all 114 cells) utilizing markers 6 (CD163) and then 1 (sialoadhesin). The resulting group contained 11 cells, 9 of which were in the SP-A1 group and 2 in the KO group. Panel D shows an additional selection from the whole cell population in which we sequentially used markers 2 (CD44), 4 (tubulin), and 10 (CD15). This resulted in a group of 13 cells, 12 of which were in the SP-A1 cohort. As in the previous samples we have marked other similar features that may be useful for describing a phenotype.

In Fig. 9 a pair of selections are demonstrated in which the first screening step involved selecting the cells below the limits given in Table 4. Panel A depicts a selection strategy in which the initial step was the selection of cells with levels of marker 10 (CD15) ***below*** the limit of ≥8. The resulting cells were then screened for levels of marker 3 (actin) at or above the limit. This search yielded 17 cells, of which 15 were in the KO group and 2 were in the SP-A1 group. In Panel B we pursued a similar strategy to select for SP-A1 cells. Our first screen was for cells with levels of marker 12 (phalloidon) ***below*** the limit of ≥8 (i.e. low levels). The resulting cells were then screened for marker 10 (CD15) at or above the limit. There were 17 cells that met these criteria. Fifteen of these were in the SP-A1 group and 2 were in the KO group.

This selection process demonstrates a method that allows us to systematically compare CMP summary data such as those shown in Fig. 6, Panel C. With this method we have identified groups of cells with similar properties that are more commonly expressed in one of our experimental groups. The observations made here indicate that despite their similarities, in a strict sense, the individual cells of either group are heterogeneous, so that no single cell is identical to another. However, the systematic comparison of CMPs by positive or negative selection enabled the identification of signatures that were predominant in one group (i.e. KO) or another (SP-A1) indicating that there is not such a thing as a clear cut (100%) division between groups of cells. Furthermore, with this method we were able to determine which of the two groups exhibited lower cellular heterogeneity by studying CMP consistency among samples of a given group.

## Discussion

In this study we investigated the effect of SP-A1 on the toponome of AM as defined by the topography of 11 proteins. We also studied cellular autofluorescence, which was granular in nature and potentially localized in lysosomes and/or phagosomes, as well as phalloidin, a marker of filamentous actin (Table 2). We did this using TIS, an advanced fluorescence microscopic system, to study for the first time, a large number of individual cells and compare their toponomic characteristics between two experimental groups. Using the CMPs generated and by applying TIS software to the images, a remarkable phenotypic diversity/heterogeneity was revealed among the AM, where no two cells (out of the 114 examined) were identical. Moreover, CMP-based categorization of these 13 markers enabled identifying molecular signatures that could not only identify cell subpopulations within the same group, but also distinguish between AM from lung of KO vs. SP-A1 mice. Our findings from this study using TIS and 13 markers were made possible because CMPs are based not simply on co-localization of proteins in cells, but also on how proteins are clustered in a cell to form supramolecular structures that are the postulated mediators of functions of proteins. Thus, similar levels of specific proteins may have very different implications on cellular function depending on the proteins present in proximity.

CMPs integrate in the toponome, which combines aspects of the *proteome* and the *interactome,* and this study reflects the assembly and/or interactions of the 13 markers in a given cellular space in intact cells. As pointed out in the Background, the AM cell population is known to have a high degree of phenotypic diversity [12, 31, 32, 50]. Hence the finding of heterogeneity identified in this study is, in itself, not surprising. What is novel, however, is the degree of heterogeneity of AMs that could be identified with just 13 markers showing that no two cells are identical, as well as the ability to characterize individual AM cells based on similarities in their CMPs (Figs. 8 and 9). Moreover, in spite of this heterogeneity, CMP signatures for each group were discerned.

When data were analyzed based on the number and/or the composition of CMPs, we noted the following about our AM populations: First, we observed that the CMPs from KO and SP-A1 were not only significantly different, but the cells from the KO mice showed significantly more conservation of CMPs (i.e. presence of identical CMPs in all members of the group) among the three mice within the group (Table 3) than the SP-A1 mice. This indicates that the KO mice and their cells exhibit greater similarity to one another than those from the SP-A1 rescue group. Conversely, SP-A1 appears to introduce more cellular diversity. The mechanisms responsible for the homogeneity/heterogeneity and/or its functional consequences are unknown. However, it has been shown that a single dose of SP-A, such as the one administered here, has a multitude of system-wide effects on the AM [7, 21, 22, 26, 30], and that its functional consequences include increased survival of mice infected with *K. pneumoniae* [29]. Thus, the absence of SP-A in the KO mice results in AM that are differentiated/activated to a lesser degree, and therefore more uniform as shown by CMP analysis (Table 3), than the KO animals rescued with SP-A1. This is consistent with previous findings where the cell size of the KO was smaller than that of the KO that had been rescued with SP-A [7, 22]. Moreover, the effect of a single dose of SP-A1 was evident within 18 h on the AM toponome, as we have demonstrated previously on the AM proteome within the same time frame [22, 26]. Together these observations support a role for SP-A in the generation of various AM subgroups. The proteomic studies gave us information about increases or decreases in the expression of specific proteins due to SP-A exposure, but because the AM are disrupted, they are unable to tell us whether the changes are in all AM or in specific subpopulations of cells. The present study presents an important advance over previous studies because it allows us to study intact cells and define these subgroups.

Secondly, when we relaxed the stringency to look at similarities between the expression of pairs of markers rather than the identity of all thirteen markers in specific CMPs, the differences between groups continued to be highly significant.

**Fig. 10:**
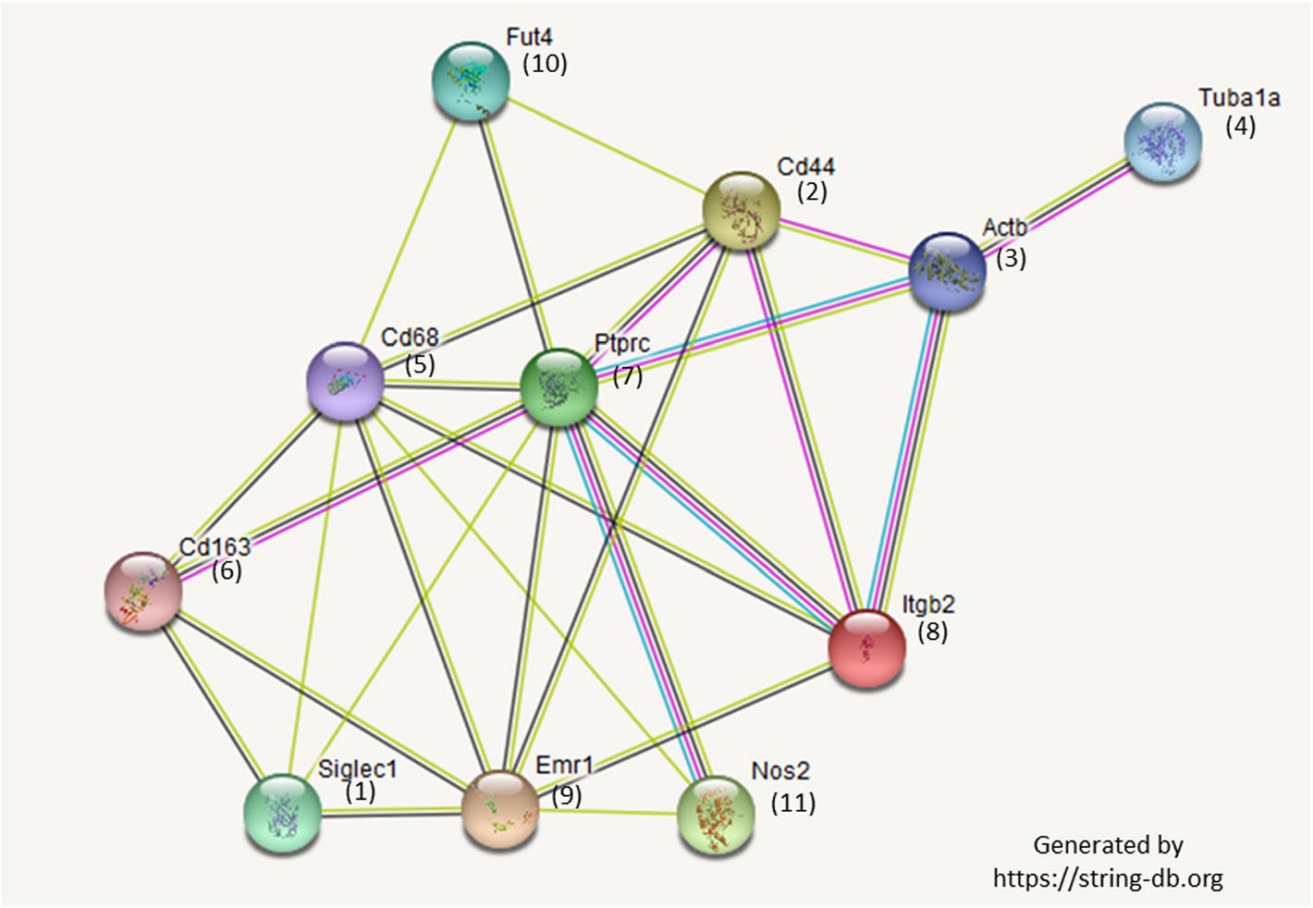
Interaction diagram. The String Database was used to generate a protein-protein interaction diagram for 11 of the markers. See Table 2 for other information on marker numbers and gene names

Third, we did an examination of a total of 114 cells by CMP analysis and showed that no two cells were identical, although groups of cells with similarities could be discerned both within a given group and between groups. Analysis of individual cells and their CMP content allowed us to define cell signatures that characterized small cell subpopulations. Subpopulations with specific signatures tended to be mostly from one experimental group or the other, indicating a dependence on either the absence of SP-A as in the KO mice, or the presence of SP-A as in the SP-A1 rescue group. There were some cell signatures/subpopulations that were not very different (not shown) between the two groups indicating that their characteristics were not dependent on SP-A. Thus, via CMP analysis we were able to identify signatures or patterns of marker expression that were predominant (although not exclusive) in one group versus the other, as well as signatures shared by both groups. The data in the present study clearly show that the division between the two groups is not an on-off switch, but most likely a rheostat where a varying predominance of certain CMP signatures are present in one group versus the other, and this may have functional consequences. In this regard SP-A1 may play a role in protein cluster organization or the formation of supramolecular structures that may underlie functional differences previously observed in response to SP-A1 [24–27]. A protein-protein interaction diagram generated by the String database (https://string-db.org) shows many of the known relationships between our selected markers (Fig. 10).

A fourth novel finding is that the less abundant CMPs tended to be composed of a greater number of the 13 markers than the more abundant CMPs, which were typically made up of fewer markers and occupied significantly more pixels (i.e. space in the cell) (Fig. 7, composition of CMPs in individual cells). This indicates that the less abundant CMPs are more diverse than the more abundant CMPs. This finding underscores the value of technologies, such as TIS, that enable identifying even rare subpopulation of cells and the limitation of technologies that provide data only on population averages. The potential use of the CMP concept to identify rare cell populations may complement and extend possibilities achieved by other microscopic multiplexing systems [51].

We postulate that under the influence of SP-A1, varying changes begin to occur in subpopulations of AM leading to a greater cell diversity, as discerned here by CMP analysis. The role of the different subpopulations of AM in innate immunity is not known and should be a subject of future investigation. However, the collective AM population with its cellular diversity could provide a broader spectrum of protection from infection or other potentially damaging stimuli as demonstrated by the improved survival of KO mice after rescue with SP-A1 [29]. This AM heterogeneity/diversity may be an adaptive mechanism for a better outcome and survival. In contrast, the CMP consistency in the KO vs. the SP-A1 group, may be linked to host defense deficits that characterize the SP-A KO mice making the KO less capable of responding to various infectious or toxic threats. We speculate that SP-A “primes” AM for a better response to various threats and KO cells, lacking this “priming,” are less differentiated and less capable of responding effectively to various noxious or infectious stimuli [22].

The relative conservation of CMPs in KO mice was seen with respect to all markers except iNOS, which is often cited as an M1 marker. On the other hand, CD68 which is also characterized as an M1 marker, showed (unlike iNOS) more consistency in the KO samples. These findings underscore the need for additional methods such as TIS to characterize AM heterogeneity and investigate the notion of the ability of SP-A to promote cellular diversity. The use of TIS to identify CMPs and thereby study the expression patterns of multiple markers and their interactions within the same cellular space provides an important first step in understanding this heterogeneity, appreciating the diversity of the AM population under various conditions, and eventually investigating its impact on different AM functions. Similar heterogeneity and phenotypic diversity is being revealed in numerous systems [52, 53] and is likely to be important for many biologically relevant systems. Our results indicate that toponomics may provide a powerful tool for exploring this phenomenon.

In addition to the information generated about the influence of SP-A on AM, an important aspect of this study was the development of approaches that allowed us to use TIS data to compare multiple samples from the same or different subjects. In previous studies TIS was used as a descriptive tool to characterize one or two samples, and in some cases to do some comparison between tissue sections from different groups of subjects [35–40, 45]. However, there has not been another study where this many individual cells (> 100) belonging to different experimental groups have been studied and compared. In this study we have, for the first time, used CMPs to directly compare AM from different experimental groups.

TIS enables localizing multiple proteins within a tissue section or intact, isolated cells on a pixel-by-pixel basis and by better preserving protein epitopes and characterizing individual AM in more detail than has previously been possible by assessing potential physical protein-protein interactions. It utilizes intact cells, so subcellular localization of the molecules being studied is preserved, which is an important advance. Proteins have a complex life cycle and pass through a number of subcellular compartments during their synthesis, post-translational modification, packaging, and trafficking to their final destination. Proteins undergoing these complex processes typically only exhibit their characteristic function when they arrive at their final destination in the cell [54]. Published studies using TIS have demonstrated the critical role that protein-protein interactions play in specific cellular functions [55].

The TIS technology used here, as noted in the Introduction, has advantages over other more recently introduced multiplexing methods [41, 51, 55, 56]. In these systems the antibodies are tagged with Cy dyes and the fluorescence is quenched by exposing the sections to H_2_O_2_ at pH > 10. We opted to use TIS for several reasons. Exposing the sections to H_2_O_2_ at pH > 10 to quench the fluorescence [41, 51, 56, 57], unlike photobleaching used in TIS, has been found to alter epitopes of some proteins and may decrease, eliminate, or enhance the fluorescence signal [41, 57]. Unlike TIS the newer systems are yet to be automated and importantly lack the capabilities offered by the image processing software developed for use with TIS and referred to above. TIS also has advantages over multi-color flow cytometric methods because it allows multiple markers to be localized within cells or subcellular compartments. Analyzing the TIS data presented considerable challenges, but we think that the approach we outlined here provides the potential to characterize cells and make comparisons between experimental groups.

The limitations of the study include: 1) The use of a single time point (18 h) after SP-A1 treatment, as we did previously with our proteomic studies [21, 22, 26]. Hence, we probably only observed the leading edge of the SP-A1 effect. However, this choice was made to keep the focus on the primary effects of SP-A1. With longer time periods, the AM molecules regulated by SP-A1 would begin to exert their own effects complicating interpretation. 2) The use of a limited number of markers. We did not include some proteins with known interactions with the markers tested or with related functions. This is because there were either no appropriate reagents available, the reagents that were tested did not give us consistent results, or we were unable to obtain artifact-free images for all 6 of our samples. 3) Although we have investigated the composition of abundant CMPs to study AM heterogeneity in the presence or absence of SP-A1, the concept of lead protein(s) (i.e. markers that are consistently present in groups of CMPs, another TIS advance), remains to be investigated with experimental designs more amenable to this type of experimentation. 4) We focused on the more abundant CMPs because we postulated that they have the greatest effect on function, but it is highly likely that the rarer, and more diverse CMPs could be equally important. 5) In this study we did not take advantage of TIS’s ability to capture and process 3D images or to deconvolute the images, processes that are required for more definitive co-localization of markers.

## Conclusions

In summary, using TIS with a panel of 13 markers to study AM from SP-A KO mice and mice treated with exogenous SP-A1: 1) we documented extensive heterogeneity/diversity of AM where no two cells are identical. 2) The AM from KO mice from all three subjects, although heterogeneous, were more uniform than those from the SP-A1 rescue group. 3) Analysis of individual cells allowed us to define cell signatures that characterized small cell subpopulations that may have functional differences. 4) Subpopulations with specific signatures were identified that tended to be mostly from one experimental group or the other indicating their potential usefulness in distinguishing cell groups shown previously to differ in several host defense functions. 5) Some CMPs were found in common between the two groups indicating that these were not dependent on SP-A1.

## Supplementary information

**Additional file 1:** The image shows the autofluorescence of the AM at the beginning of the experiment. Two cells are enlarged in the inset (lower left). The autofluorescence was completely eliminated by the standard series of photobleaching cycles. The autofluorescent signal was binarized along with the other markers and is included in the data set as marker 0.

## Data Availability

The datasets used and/or analysed during the current study are available from the corresponding author on reasonable request.

## Abbreviations

AF: Autofluorescence
AM: Alveolar macrophage
BAL: Bronchoalveolar lavage
CMP: Combinatorial molecular phenotype
FITC: Fluorescein isothiocyanate
KO: SP-A knockout
MELC: Multi-epitope ligand cartography
SP-A: Surfactant protein A
TIS: Toponome Imaging System
TOF: Time-of-flight
