# Supplementary figures and images for: Using toponomics to characterize phenotypic diversity in alveolar macrophages from male mice treated with exogenous SP-A1
Source: Biomark Res. 2020 Feb 13;8:5. doi: 10.1186/s40364-019-0181-z (PMC7020580; doi:10.1186/s40364-019-0181-z)

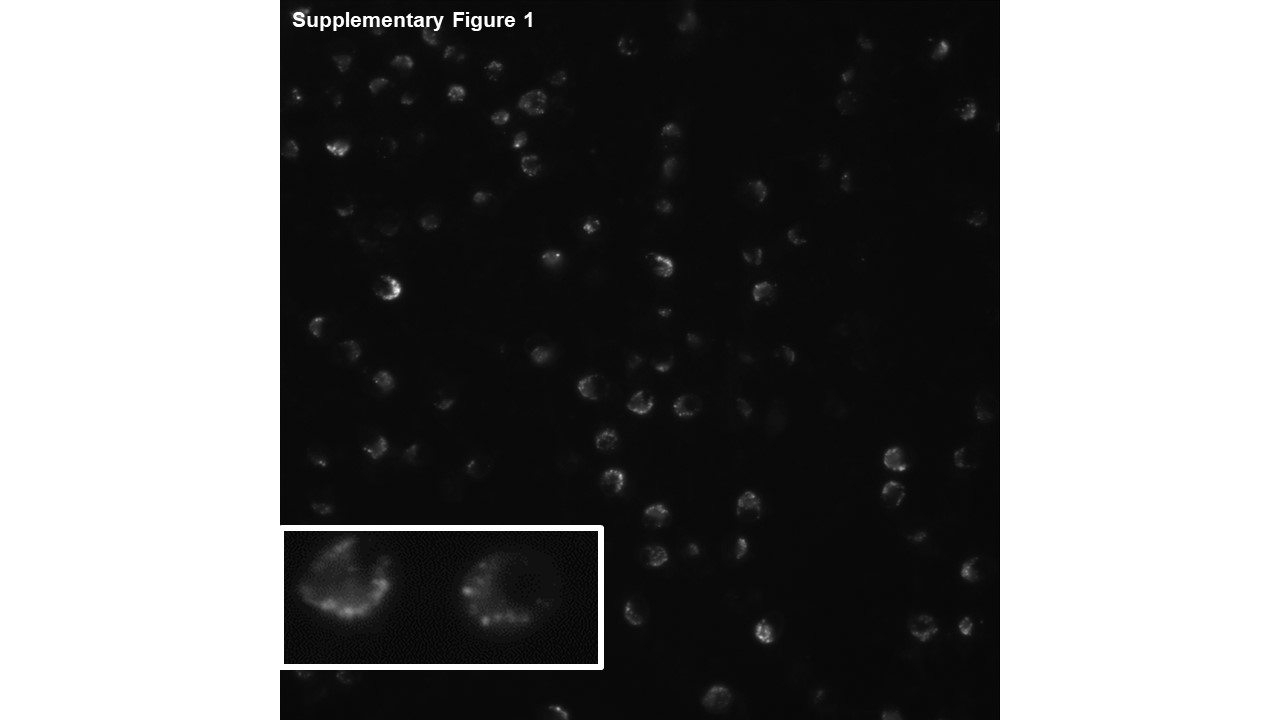

Supplement: Supplementary file 1 — Additional file 1: The image shows the autofluorescence of the AM at the beginning of the experiment. Two cells are enlarged in the inset (lower left). The autofluorescence was completely eliminated by the standard series of photobleaching cycles. The autofluorescent signal was binarized along with the other markers and is included in the data set as marker 0. [file 40364_2019_181_MOESM1_ESM.jpg]
